# Targeting enolase isoenzyme 2 enables precision glycolysis inhibition in *VHL*-mutant renal cell carcinoma

**DOI:** 10.1038/s41419-026-09102-4

**Published:** 2026-07-14

**Authors:** Jiannan Yao, Xiaona Fan, Huixian Xin, Fuhui Wan, Xichen Dong, Qianqian Sun, Xuying Huang, Heshu Liu, Xiangyu Chen, Yanjun Jia, Haili Qian, Tao Wen, Jian Liu

**Affiliations:** 1 Department of Oncology, Beijing Chao-Yang Hospital, Capital Medical University, 100020 Beijing, China; 2 Department of Gastroenterology, Beijing Chao-Yang Hospital, Capital Medical University, 100020 Beijing, China; 3 Medical Research Center, Beijing Chao-Yang Hospital, Capital Medical University, 100020 Beijing, China; 4 Department of Radiation Oncology, Qilu Hospital of Shandong University, Jinan, 250012 Shandong China; 5 State Key Laboratory of Molecular Oncology, National Cancer Center/National Clinical Research Center for Cancer/Cancer Hospital, Chinese Academy of Medical Sciences and Peking Union Medical College, 100021 Beijing, China

**Keywords:** Cancer metabolism, Cancer therapy

## Abstract

Clear cell renal cell carcinoma (ccRCC) exhibits glycolytic addiction due to VHL mutation, making it vulnerable to metabolic intervention. While conventional inhibitors targeting core housekeeping glycolytic enzymes show robust antitumor efficacy, their clinical use is limited by on-target systemic toxicity. Here, we identify enolase 2 (ENO2) as an isoform-specific glycolytic vulnerability specific to VHL-deficient ccRCC. Although ENO2 is physiologically redundant (compensated by ENO1) in normal glycolysis, it becomes essential for sustaining ccRCC malignancy via VHL loss-HIF2α-driven glycolytic flux. Targeting ENO2 specifically suppressed glycolysis in VHL-deficient ccRCC cells, reducing their malignancy and enhancing their response to axitinib, doxorubicin, and gemcitabine. Importantly, pharmacological ENO2 blockade displayed superior tumor selectivity compared to traditional glycolysis inhibitors, effectively eliminating VHL-deficient ccRCC cells while sparing VHL-intact normal and malignant cells, despite causing slightly weaker glycolytic suppression. Mechanistic investigations revealed that ENO2 ablation inactivated up to ~78% of VHL loss-induced oncogenic effectors, including a pleiotropic oncoprotein MDK, through dual modulation of lactate metabolism and interferon signaling. Collectively, this work reveals ENO2 as a genotype-specific metabolic dependency in ccRCC, thereby enabling precision glycolytic therapy. Our work also suggests that targeting physiologically redundant metabolic isoenzymes may offer a precision medicine strategy for cancers with defined genetic alterations.

## Introduction

Clear cell renal cell carcinoma (ccRCC) accounts for approximately 75% of all renal malignancies and is a prototypical metabolic disorder characterized by a pronounced dependence on glycolysis [1–7]. This metabolic feature arises from the mutation and functional inactivation of the von Hippel-Lindau (*VHL*) tumor suppressor gene in over 90% of ccRCC cases, causing the constitutive stabilization of hypoxia-inducible factors (HIFs) and subsequent metabolic reprogramming [1, 7, 8]. Such metabolic rewiring not only fuels tumor growth, angiogenesis, and therapeutic resistance, but also exposes a pivotal vulnerability for targeted therapy [5, 9].

While conventional glycolytic inhibition strategies targeting key enzymes (e.g., HK2, PFKFB3, LDHA, ENO1) demonstrate potent antitumor effects, their clinical translation is limited by poor tumor specificity and systemic toxicity [10–12]. This limitation arises from the essential housekeeping roles these enzymes play in normal cellular metabolism, resulting in inherent on-target toxicity in healthy tissues when systemically inhibited, which disrupts critical physiological processes [11, 13, 14].

Recent studies reveal that cancers often hijack specific isoenzymes to fulfill heightened metabolic demands [15–18]. Identifying these isoenzymes, particularly those with redundant roles in physiological metabolism, offers a promising approach for developing tumor-selective glycolytic inhibition strategies.

Our current investigation reveals that enolase isoenzyme 2 (ENO2), although functionally redundant in normal glycolysis, emerges as a ccRCC-specific metabolic dependency in the context of VHL deficiency. The enolase family comprises three distinct isoenzymes (ENO1, ENO2, and ENO3) that catalyze the conversion of 2-phosphoglycerate to phosphoenolpyruvate in the penultimate step of glycolysis [19, 20]. Among these isoforms, ENO1 is ubiquitously high-expressed and serves essential housekeeping functions, whereas ENO3 displays muscle-specific expression and activity [21, 22]. In contrast, ENO2 is minimally expressed in most normal tissues, with elevated levels observed only in neuronal and neuroendocrine tissues[23]. Importantly, ENO2 demonstrates functional redundancy in normal physiological contexts due to compensatory ENO1 activity [21, 22]. This redundancy is evidenced by preclinical studies showing that pharmacological inhibition of ENO2 causes no observable toxicity, even in neural tissues [22, 24]. The enzyme becomes indispensable only under rare pathological conditions, such as ENO1-deficient gliomas [22, 24].

We demonstrate here that the physiologically redundant ENO2 serves as the pivotal enolase isoform enabling metabolic adaptation in *VHL*-mutated ccRCC. Pharmacological or genetic inhibition of ENO2 selectively impairs glycolytic flux and cellular viability in VHL-deficient tumors while exhibiting minimal effects on normal cells and VHL-wildtype malignancies. This isoform-specific vulnerability stems from ENO2’s unique regulation by the VHL-HIF2α axis and its indispensable role in supporting ccRCC’s metabolic reprogramming. These findings identify ENO2-targeted therapy as a precision metabolic intervention for ccRCC, providing both a mechanistic framework and therapeutic strategy for genotype-selective cancer treatment.

## Materials and methods

### Sex as a biological variable

Our study exclusively examined male mice. It is unknown whether the findings are relevant for female mice.

### Ethics statement

All procedures were conducted in accordance with the ethical standards of the responsible committees on human and animal research. Human tissue studies were approved by the Ethics Committee of Beijing Chao-Yang Hospital, Capital Medical University (No. 2021-ke-676) with written informed consent from all participants. Animal experiments were approved by the Laboratory Animal Welfare and Ethics Committee of Capital Medical University (No. AEEI-2021-316) and complied with all relevant institutional and national guidelines.

### Cell culture

All cell lines used in this study were obtained from the National Collection of Authenticated Cell Cultures (Beijing, China) or other specified reliable sources. VHL-deficient ccRCC cell lines (786-O, 769-P, and OSRC-2) were cultured in RPMI-1640 medium (Sigma, Austria) supplemented with 10% fetal bovine serum (FBS, Ausbian, Australia) and 1% penicillin-streptomycin (P/S) (Gibco, USA). VHL-wt Caki-1 and Caki-2 cells were maintained in McCoy’s 5 A medium (KeyGEN BioTECH, China), while VHL-wt ACHN cells were cultured in Eagle’s Minimum Essential Medium (EMEM; MeilunBio, China). QGP neuroendocrine tumor cell line was cultured in RPMI-1640 medium (Gibco, USA). Normal cell lines were obtained from Haili Qian Laboratory (State Key Laboratory of Molecular Oncology, National Cancer Center/National Clinical Research Center for Cancer/Cancer Hospital, Chinese Academy of Medical Sciences and Peking Union Medical College, Beijing, China). HK-2 was cultured in DMEM/F12 (Procell, China) and HEK-293T in high-glucose DMEM (Gibco, USA). HIEC cells were maintained in Opti-MEM supplemented with 4% FBS and 20 ng/mL EGF. MCF10A cells were grown in DMEM/F12 with 5% horse serum, 20 ng/mL EGF, 10 μg/mL insulin, 0.5 μg/mL hydrocortisone, and 100 ng/mL cholera toxin. 16HBE and Beas-2B cells were cultured in BEGM (Lonza, Switzerland). Unless otherwise specified, all media were supplemented with 10% FBS (Ausbian, Australia) and 1% P/S, (Gibco, USA). All cell lines were incubated at 37 °C in a humidified incubator with 5% CO₂. Cells were passaged at 70–90% confluence using 0.25% trypsin-EDTA (Gibco, USA) and routinely tested for mycoplasma contamination by PCR.

### Clinical patient samples

The study protocol was approved by the Ethics Committee of Beijing Chao-Yang Hospital, Capital Medical University, and conducted in accordance with the Declaration of Helsinki. Written informed consent was obtained from all patients before sample collection. Paired tumor and adjacent non-tumor kidney tissues were collected from patients with histologically confirmed clear cell renal cell carcinoma who underwent nephrectomy at Beijing Chao-Yang Hospital. Tissue specimens were snap-frozen in liquid nitrogen immediately after surgical resection and stored at −80 °C. Additionally, a cDNA microarray (product number: cDNA-HKidE030CS01) containing paired cDNA samples from 15 ccRCC patients was purchased from Shanghai Outdo Biotech.

### Protein extraction and Western blotting

Total protein was extracted from tissues and cultured cells using RIPA lysis buffer (Solarbio, China) supplemented with PMSF protease inhibitor (Beyotime, China). Approximately 50 mg of tumor or adjacent normal tissue was homogenized on ice in ice-cold RIPA buffer using a tissue homogenizer (Thermo Fisher Scientific, USA). Cultured cells were lysed by directly adding cold RIPA buffer to the monolayers. Lysates were centrifuged at 12,000 × *g* for 20 min at 4 °C, and supernatants were collected. Protein concentration was quantified using the BCA Protein Assay Kit (Thermo Fisher Scientific, USA) following the manufacturer’s protocol. Equal amounts of protein (40 μg) were denatured with 5× SDS-PAGE loading buffer (Yeasen, China), boiled at 95 °C for 10 min, and separated on 7.5–15% SDS-PAGE gels prepared using a PAGE gel preparation kit (Epizyme Biotech, China). Proteins were then transferred to 0.2 μm PVDF membranes (Millipore, USA) using a wet or semi-dry transfer apparatus. After transfer, membranes were blocked, incubated with primary antibodies overnight at 4 °C, and then with secondary antibodies. Details of all antibodies, including sources, dilutions, and catalog numbers, are listed in Supplementary Table 1. Signal detection was conducted using ECL reagent (Millipore, USA), and images were acquired with the BG-gdsAUTO 710 MINI imaging system (Baygene, China). Protein band intensities were quantified using ImageJ software (NIH, USA). Relative protein expression levels were normalized to internal loading controls.

### RNA extraction and quantitative RT-PCR analysis

Total RNA was extracted using Trizol TRIeasy Reagent (Yeasen Biotechnology, China) according to the manufacturer’s instructions. RNA concentration and purity were assessed using a NanoDrop spectrophotometer (Thermo Fisher Scientific, USA). Only samples with an A260/A280 ratio between 1.8 and 2.0 were used for subsequent analysis. Reverse transcription and RT-qPCR were performed using the One-Step RT-qPCR Kit (Accurate Biotechnology, China) following the manufacturer’s instructions. PCR primers were synthesized by Sangon Biotech (Shanghai, China), and sequences for primers and probes targeting specific genes and the internal control (β-actin) are listed in Supplementary Table 2. All RT-qPCR assays were performed on an ABI 7500 Real-Time PCR System (Applied Biosystems, USA). Relative mRNA expression levels were calculated using the 2^ − ΔΔCT method, with β-actin as the internal control.

### Gene overexpression and knockout

Lentiviral-mediated overexpression was used to establish stable cell lines expressing ENO2, VHL, HIF-2α, or MDK. Full-length coding sequences were cloned into lentiviral vectors containing a puromycin resistance gene for selection. These plasmids and corresponding empty vector controls were obtained from UBIGENE (Guangzhou, China). For lentiviral packaging, HEK293T cells were co-transfected with target plasmids and packaging vectors (psPAX and pMD2.G) using Lipofectamine 3000 (Invitrogen, USA). Viral supernatants were collected at 48 and 72 h post-transfection and filtered through 0.45 μm syringe filters before use. Target cells were seeded in 6-well plates (2 × 10⁵ cells/well) and incubated overnight. The next day, cells were transduced with viral particles in 8 μg/mL Polybrene (Beyotime, China) for three consecutive days. Puromycin (2 μg/mL, Beyotime, China) was used for 7–10 days to select stable cell lines, with medium changed every 48 h.

The CRISPR/Cas9 system was employed to establish ENO2-deficient cell lines. Three guide RNAs (sgRNAs) were designed and cloned into the lentiCRISPR v2 vector. Lentiviral particles were packaged, transduced, and selected using the same procedures as for overexpression. Knockout efficiency was verified by Western blotting. The sgRNA sequences targeting ENO2 were:

ENO2-sgRNA-1: Oligo1 5′-CACCGCCAACCCAAAACGTATTGAG-3′ Oligo2 3′-CGGTTGGGTTTTGCATAACTCCAAA-5′

ENO2-sgRNA-2: Oligo1 5′-CACCGGGGAGATCCTGGACTCCCGC-3′ Oligo2 3′-CCCCTCTAGGACCTGAGGGCGCAAA-5′

ENO2-sgRNA-3: Oligo1 5′-CACCGGATCTTCTCTATGGACATGA-3′ Oligo2 3′-CCTAGAAGAGATACCTGTACTCAAA-5′

### SiRNA-mediated HIFα knockdown

Small interfering RNAs (siRNAs) targeting HIFα or HIF2α (Sangon Biotech) were used to transiently silence HIF1α or HIF2α expression in the indicated cells. The siRNA sequences were as follows:

HIF1α siRNA: 5′-GCGAAGUAAAGAAUCUGAA [dT][dT]-3′;

HIF2α siRNA: 5′-GCCGUACUGUCAACCUCAA [dT][dT]-3′.

A non-targeting siRNA was used as the negative control. Cells were transfected with the indicated siRNAs using Lipofectamine RNAiMAX reagent (Invitrogen, Carlsbad, CA, USA) according to the manufacturer’s instructions.

### Seahorse XF glycolysis stress test assay

The Seahorse XF Glycolysis Stress Test was used to evaluate the extracellular acidification rate (ECAR), a surrogate of glycolytic activity. Cells were seeded in Seahorse XF96 cell culture microplates (Agilent Technologies, USA) at a density of 1 × 10⁴ cells/well and incubated overnight at 37 °C in a humidified incubator with 5% CO₂. On the assay day, the culture medium was replaced with Seahorse XF assay medium (Agilent Technologies, USA) supplemented with 2 mM glutamine (Agilent Technologies, USA), and cells were incubated for 1 h at 37 °C in a non-CO₂ incubator for pH equilibration. ECAR was monitored in real time using the Seahorse XF96 Analyzer (Agilent Technologies, USA). The assay included sequential injections of 10 mM glucose, 1 µM oligomycin, and 50 mM 2-deoxyglucose (2-DG) to assess basal glycolysis, glycolytic capacity, and glycolytic reserve. All procedures were performed according to the manufacturer’s protocol. Data were normalized to cell number and analyzed using Seahorse Wave software (Agilent Technologies, USA).

### Glucose uptake assay using 2-NBDG

Glucose uptake was measured using the fluorescent glucose analog 2-NBDG. Cells were seeded in 6-well plates (2 × 10⁵ cells/well) and cultured overnight under standard conditions. After two PBS washes (Solarbio, China), cells were incubated in glucose-free DMEM (Gibco, USA) for 1 h at 37 °C to enhance glucose uptake sensitivity. Cells were then incubated with 100 μM 2-NBDG (Selleck, USA) in glucose-free medium for 30 min at 37 °C in the dark. After incubation, cells were washed three times with ice-cold PBS, trypsinized, and resuspended in PBS. Fluorescence intensity was analyzed immediately using flow cytometry (BD FACSCanto II, USA) at excitation/emission wavelengths of 488/530 nm. Data were processed using FlowJo software (v10.6), and mean fluorescence intensity (MFI) was used to quantify glucose uptake. All experiments were conducted in biological triplicates.

### Lactate concentration measurement

Lactate concentrations in cell culture media were quantified using a colorimetric assay to assess glycolytic output under various experimental conditions. Cells were seeded in 6-well plates (2 × 10⁵ cells/well, 2 mL medium) and cultured at 37 °C in a humidified incubator with 5% CO₂ until reaching 70–80% confluence. Cells were then subjected to treatments including ENO2 knockout (ENO2-KO) or pharmacological inhibition using glycolytic inhibitors such as 2-DG (5 mM), oxamate (40 mM), AP-III-a4 (10 μM), SF2312 (350 μM), and POMHEX (300 nM). DMSO-treated and non-targeting control (NC) cells served as vehicle and genetic controls, respectively. After treatment, culture medium was replaced with fresh complete medium, and cells were incubated for another 24 h. Supernatants were collected, centrifuged at 1000 × *g* for 10 min to remove debris, and stored at −80 °C for later analysis. Lactate levels were measured using a commercial assay kit (Sigma-Aldrich, USA) following the manufacturer’s protocol. Briefly, 50 μL of culture medium was mixed with assay buffer and reaction mix, incubated at room temperature for 30 min, and absorbance was read at 570 nm using a microplate reader (Thermo Fisher Scientific, USA). Lactate concentrations were calculated using a standard curve generated with known lactate concentrations. All measurements were conducted in biological triplicates.

### ATP quantification assay

Intracellular ATP levels were measured using a luciferin–luciferase bioluminescence assay (ATP Determination Kit, Molecular Probes, USA). Cells were seeded in 6-well plates (2 × 10⁵ cells/well) and cultured overnight under standard conditions. For pharmacological inhibition, cells were treated with glycolytic inhibitors—2-DG (5 mM), oxamate (40 mM), AP-III-a4 (10 μM), SF2312 (350 μM), and POMHEX (300 nM)—for 4 h. ENO2-KO and non-targeting control (NC) cells were used to compare ATP levels under identical treatment conditions. After treatment, cells were washed with ice-cold PBS and lysed in ATP-free lysis buffer (200 μL per well). Lysates were collected and maintained on ice. Ninety microliters of freshly prepared reaction buffer—composed of 0.5 mM D-luciferin, 1.25 μg/mL firefly luciferase, 25 mM Tricine (pH 7.8), 5 mM MgSO₄, 100 μM EDTA, and 1 mM DTT—was added to 10 μL of lysate in a 96-well opaque plate. Luminescence was recorded immediately using a microplate luminometer (BioTek Synergy H1), and ATP levels were calculated from a standard curve generated using ATP standards (1 nM–1 μM). All measurements were conducted in biological triplicates. To prevent degradation, all steps were conducted under ATP-free conditions, protected from light, and completed within 10 min post-lysis.

### Cell proliferation assay

Cell proliferation was evaluated using the Cell Counting Kit-8 (CCK-8; Lablead, China) following the manufacturer’s instructions. Cells were seeded in 96-well plates (2 × 10³ cells/well in 200 µL complete medium with 10% FBS) and incubated overnight under standard conditions (37 °C, 5% CO₂). At specified time points, the medium was replaced with 100 µL of serum-free medium, and 10 µL of CCK-8 reagent was added to each well. Plates were incubated at 37 °C for 1–2 h in the dark. Absorbance at 450 nm was measured using a microplate reader (Thermo Fisher Scientific, USA), serving as an indirect indicator of viable cell numbers.

### Transwell migration and invasion assays

Migration and invasion assays were conducted using 24-well Transwell chambers with 8.0 μm pore-size polycarbonate membranes (Corning, USA). For invasion assays, upper chambers were pre-coated with 50 µL of Matrigel® (1:8 dilution in serum-free medium) and incubated at 37 °C for 2 h to allow gel solidification. For both assays, 200 µL of cell suspension (5 × 10⁴ cells in serum-free medium) was added to the upper chambers, and 700 µL of complete medium with 10% FBS was placed in the lower chambers as a chemoattractant. Plates were incubated at 37 °C with 5% CO₂ for 24 h (migration) or 48 h (invasion). After incubation, non-migrated or non-invaded cells on the upper membrane surface were gently removed using a cotton swab. Membranes were washed with PBS, fixed in 4% paraformaldehyde (20 min, room temperature), and stained with 0.1% crystal violet (15 min). After staining, membranes were rinsed with distilled water, and migrated or invaded cells on the lower membrane surface were imaged using an Olympus SZX16 stereo microscope (Olympus, Japan) in bright-field mode at ×40 magnification. Migrated or invaded cells were manually counted.

### Apoptosis assay

Apoptosis was assessed by flow cytometry using the Annexin V-FITC/PI Apoptosis Detection Kit (KeyGen Biotech, China), according to the manufacturer’s instructions. Cells were harvested after treatment, washed twice with cold PBS, and resuspended in 100 µL of binding buffer at 1 × 10⁶ cells/mL. Annexin V-FITC (5 µL) and PI (5 µL) were added, and samples were incubated in the dark for 15 min at room temperature. Following staining, 400 µL of binding buffer was added, and samples were analyzed within one hour using a BD FACSCanto II flow cytometer (BD Biosciences, USA). Apoptotic cell populations were identified: early apoptotic (Annexin V⁺/PI⁻), late apoptotic (Annexin V⁺/PI⁺). Apoptotic populations were quantified using FlowJo software (v10.7.1, FlowJo LLC, USA).

### Tube formation assay

Tube formation assays were conducted to assess the angiogenic potential of HUVECs in response to conditioned media from renal carcinoma cells. LDEV-free Corning® Matrigel® (Corning, USA) was thawed overnight at 4 °C and added to 96-well plates (50 µL/well). Plates were incubated at 37 °C for 30 min to allow polymerization. HUVECs were seeded on solidified Matrigel (1 × 10⁴ cells/well) in endothelial growth medium containing 10% FBS and 1% P/S. Conditioned media were centrifuged to remove debris and used to replace the standard growth medium. Cells were incubated at 37 °C with 5% CO₂ for 6–12 h to allow capillary-like structure formation. Tubular structures were imaged using an Olympus IX73 inverted microscope (Olympus, Japan) at 10× magnification. Representative images were taken from three independent fields per well, and experiments were performed in triplicate.

### VHL-loss selectivity index (VSI) calculation

The VHL-loss Selectivity Index (VSI) was calculated to quantify the effect of VHL status on renal cancer cell sensitivity to metabolic inhibitors, based on relative inhibition in VHL-deficient (VHL–) and VHL-rescued (VHL + ) cells. The inhibition rate (IR) was defined as the percent reduction in metabolic readouts (e.g., ATP or lactate) relative to the negative control: IR = (1—treated group / NC group) × 100%. VSI was calculated as the ratio of IR in VHL-deficient cells to that in VHL-restored cells: VSI = IR(VHL − ) / IR(VHL + ). A VSI > 1 indicates greater sensitivity in VHL-deficient cells; VSI < 1 indicates greater sensitivity in VHL-rescued cells; VSI ≈ 1 suggests no significant difference. All measurements were conducted in biological triplicates, and mean values were used for analysis. This approach was applied to both ATP-based viability and lactate production assays to assess the metabolic effects of inhibitors in different VHL contexts.

### Drug sensitivity assay and IC_50_ determination

Cell sensitivity to metabolic inhibitors was assessed using the Cell Counting Kit-8 (CCK-8; Lablead, China), following the manufacturer’s instructions. Briefly, cells were seeded in 96-well plates (2 × 10³ cells/well in 200 µL complete medium with 10% FBS) and incubated overnight at 37 °C in a humidified incubator with 5% CO₂. The next day, the medium was replaced with fresh complete medium containing serial drug dilutions to generate dose–response curves. After 48 or 72 h of incubation, depending on the experimental design, 10 µL of CCK-8 was added to each well and incubated at 37 °C for 1–2 h. Absorbance at 450 nm was measured using a microplate reader (Thermo Fisher Scientific, USA). Cell viability was calculated as a percentage relative to untreated control wells. IC₅₀ values were derived using nonlinear regression analysis in GraphPad Prism (v8.4.0).

### Tumor xenograft assay

All animal experiments were approved by the Institutional Animal Care and Use Committee of Capital Medical University (Beijing, China). Mice were obtained from Charles River Laboratories (Beijing, China) and maintained under specific pathogen-free (SPF) conditions. Three 786-O xenograft models—subcutaneous cell injection, subcutaneous tumor xenograft seeding, and orthotopic cell injection—were used to assess the impact of ENO2 depletion on tumor growth. Cells were cultured to 70–80% confluence, harvested, washed with PBS, and resuspended at 2 × 10⁷ cells/mL. For subcutaneous injection, 2 × 10⁶ cells were injected into the right flank of 4-week-old male BALB/c nude mice. For xenograft seeding, tumors were excised, cut into ~2 × 2 × 2 mm³ fragments, and implanted subcutaneously into the right flank. Tumor volumes were measured every 3 days with digital calipers and calculated as V = (length × width²)/2. Mouse body weight and health were monitored throughout. At the endpoint, mice were euthanized, and tumors were collected, imaged, and weighed. For orthotopic injection, mice were anesthetized with isoflurane and placed in lateral decubitus position. A ~ 0.5 cm incision was made in the left flank to expose the kidney, and 2 × 10⁶ cells (20 µL) were injected into the subcapsular space using a 29 G insulin syringe. At the endpoint, both tumor-bearing and contralateral kidneys were collected for analysis. To evaluate the effect of ENO2 deletion on axitinib efficacy in vivo, mice with similarly sized 786-O xenografts were randomized into four groups (*n* = 6), and axitinib (Sigma-Aldrich, USA) was administered daily by oral gavage. To evaluate the in vivo therapeutic efficacy of POMHEX, mice harboring subcutaneous 786-O xenografts of comparable sizes were randomly assigned to either the vehicle control or POMHEX treatment group. Treatment was initiated on day 32 post-tumor implantation. Mice in the treatment group received POMHEX at a dose of 15 mg/kg body weight via intraperitoneal injection every other day, while control mice were administered an equal volume of vehicle. Body weight and general condition were monitored throughout the study to evaluate treatment tolerability.

### Transcriptome sequencing and functional enrichment

Total RNA was extracted and sequenced using the Illumina PE150 platform. Sequencing reads were aligned to the human genome (hg38) using STAR (v2.7.10a), and differentially expressed genes (DEGs) were identified using DESeq2 (v1.36.0). Gene Ontology (GO) enrichment analysis was performed using the clusterProfiler package. Gene symbols were converted to Entrez IDs using org.Hs.eg.db, and significantly enriched GO terms (adjusted *p* < 0.05) were visualized as bubble plots using enrichplot. Gene Set Enrichment Analysis (GSEA) was conducted on DEGs using interferon-alpha and interferon-gamma response gene sets from the MSigDB database (v7.5.1).

### Bioinformatic analysis

Gene expression profiles of ENO1, ENO2, and ENO3 in normal human tissues were retrieved from the GTEx Portal (https://gtexportal.org). TPM-normalized RNA-seq data were visualized using R (v4.2.2) with reshape2, ggplot2, and pheatmap. Transcriptomic data for ccRCC, GBM, and LGG were obtained from TCGA via direct download or online platforms such as GEPIA2 (http://gepia2.cancer-pku.cn/) and GSCA (http://bioinfo.life.hust.edu.cn/GSCA/). Differential expression was assessed using Wilcoxon rank-sum tests or built-in statistical modules, with visualizations in box or bubble plots. The prognostic significance of ENO1 and ENO2 in TCGA-KIRC was analyzed using GEPIA2. MDK expression was evaluated by stratifying patients into high and low expression groups. Kaplan–Meier analysis was conducted using survfit() from the survival package (v3.5.7) and visualized via survminer (v0.4.9); significance was determined by the log-rank test. ENO2 and MDK expression correlation was analyzed using log₂ TPM data from TCGA-KIRC via UCSC Xena (https://xena.ucsc.edu/). Spearman correlation was computed using corr.test() (psych v2.3.6) and visualized with ggplot2 (v3.4.2), including 95% confidence intervals. Associations between ENO2 expression and *VHL* genomic alterations (CNA and mutations) were examined using cBioPortal (https://www.cbioportal.org/).

### Statistical analysis

Data analysis was conducted using R (version 4.2.2) or GraphPad Prism (version 8.4.0), unless otherwise specifically indicated. Two-group comparisons were performed using two-tailed Student’s *t* test or Wilcoxon rank-sum test, and one-way ANOVA with Dunnett’s post hoc test was used for multiple comparisons. All experiments were conducted in biological triplicates, and data are expressed as mean ± standard deviation (SD). A *p* value < 0.05 was considered statistically significant. *p < 0.05, **p < 0.01, ***p < 0.001, ****p < 0.0001.

## Results

### ENO2, the physiologically redundant enolase isoenzyme, is selectively upregulated in ccRCC

**Fig. 1:**
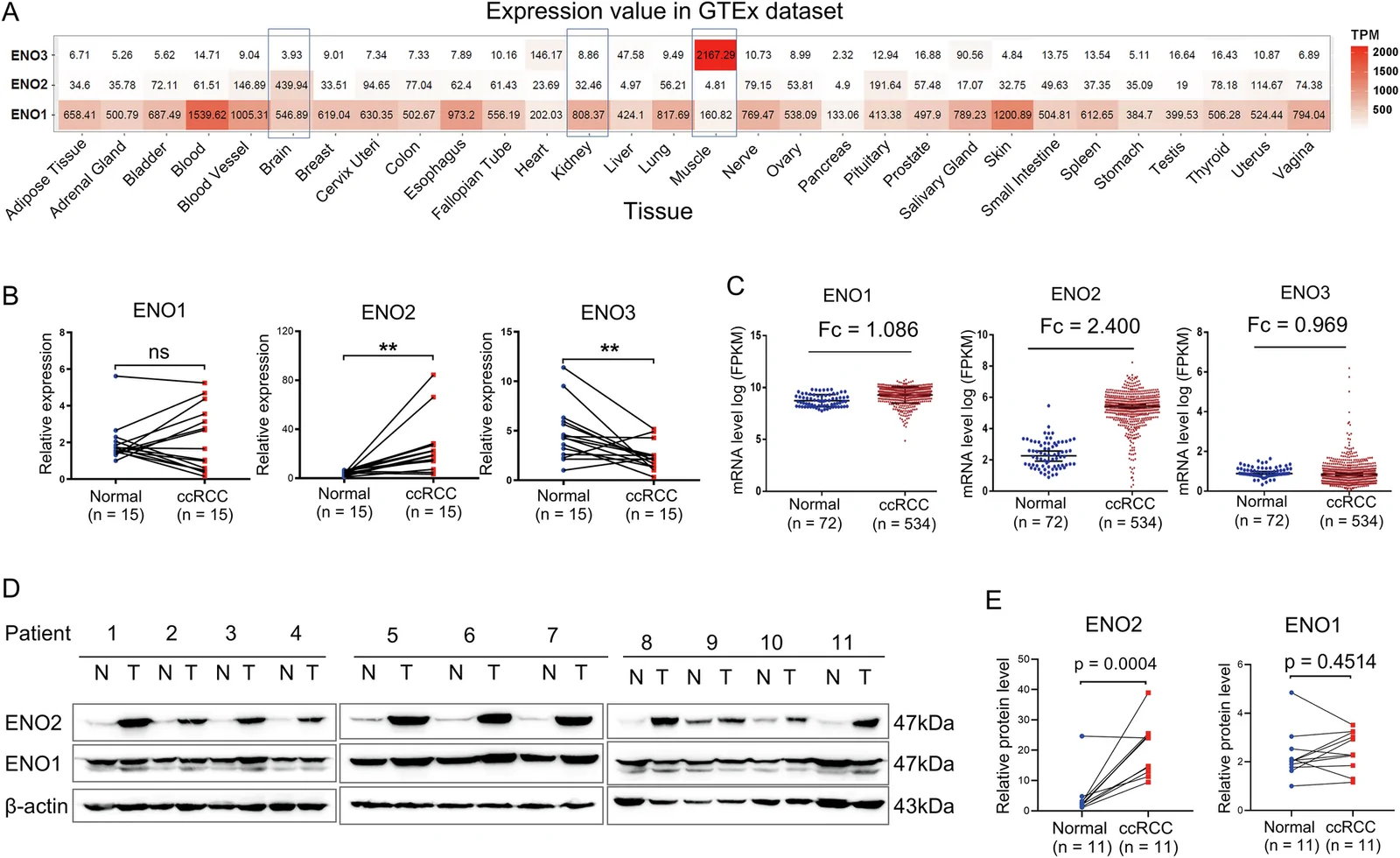
ENO2, the physiologically redundant enolase isoenzyme, is selectively upregulated in ccRCC. **A** Expression of ENO1, ENO2, and ENO3 across normal human tissues in the GTEx dataset. **B** cDNA microarray analysis of ENO1, ENO2, and ENO3 expression in matched normal kidney and ccRCC tissues from 15 patients. **C** TCGA analysis comparing ENO1, ENO2, and ENO3 mRNA expression levels between adjacent normal kidney (*n* = 72) and ccRCC tissues (*n* = 534). Fold-change (Fc) values are indicated. **D** Representative western blot showing ENO1 and ENO2 expression in paired normal (N) and tumor (T) tissues from 11 ccRCC patients. **E** Quantification of ENO1 and ENO2 protein levels in paired samples from (D) (*n* = 11). Data were analyzed by paired two-tailed Student’s *t* test.*p < 0.05, **p < 0.01, ***p < 0.001, ****p < 0.0001; ns not significant.

Supporting the established roles of ENO1 as the housekeeping enolase isoenzyme and ENO2 as the redundant isoenzyme in physiological glycolysis [21–23], GTEx analysis demonstrates that ENO1 is highly expressed across all normal human tissues, whereas ENO2 exhibits low basal expression in most tissues, except a moderate enrichment in neural tissues (Fig. 1A). Notably, even in the brain, where ENO2 expression is moderate, ENO1 maintains high expression (Fig. 1A), explaining the viability of ENO2 knockout in neuronal cells and the lethality observed only upon ENO2 knockout in ENO1-deficient neurons [22, 24]. Consistent with prior reports [21, 22], GTEx data confirm that ENO3 is exclusively highly expressed in skeletal muscle (Fig. 1A).

Assessment of enolase expression via cDNA microarray revealed significant upregulation of ENO2 in ccRCC, with no notable change in ENO1 and a mild downregulation of ENO3 (Fig. 1B). This pattern was corroborated by TCGA analysis, which identified ENO2 as the most significantly upregulated enolase isoenzyme in ccRCC (Fig. 1C). Western blot analysis of 11 paired clinical ccRCC specimens further confirmed pronounced ENO2 upregulation (Fig. 1D, E). ENO1 levels remained stable (Fig. 1D–E), while ENO3 was undetectable.

In contrast to its marked upregulation in ccRCC, ENO2 is largely downregulated in neurological tumors—including glioblastoma multiforme (GBM) and brain lower grade glioma (LGG)—relative to normal tissues (Fig. S1). This downregulation further supports prior evidence that ENO2 is dispensable for neuronal cell survival when ENO1 functionality is intact [22, 24].

Collectively, these findings indicate that ENO2, which is normally low-expressed and functionally redundant in normal tissues, undergoes selective upregulation in ccRCC, highlighting its potential role as a key enolase isoenzyme in the glycolytic rewiring of this malignancy.

### VHL loss drives ENO2 activation via HIF2α

**Fig. 2:**
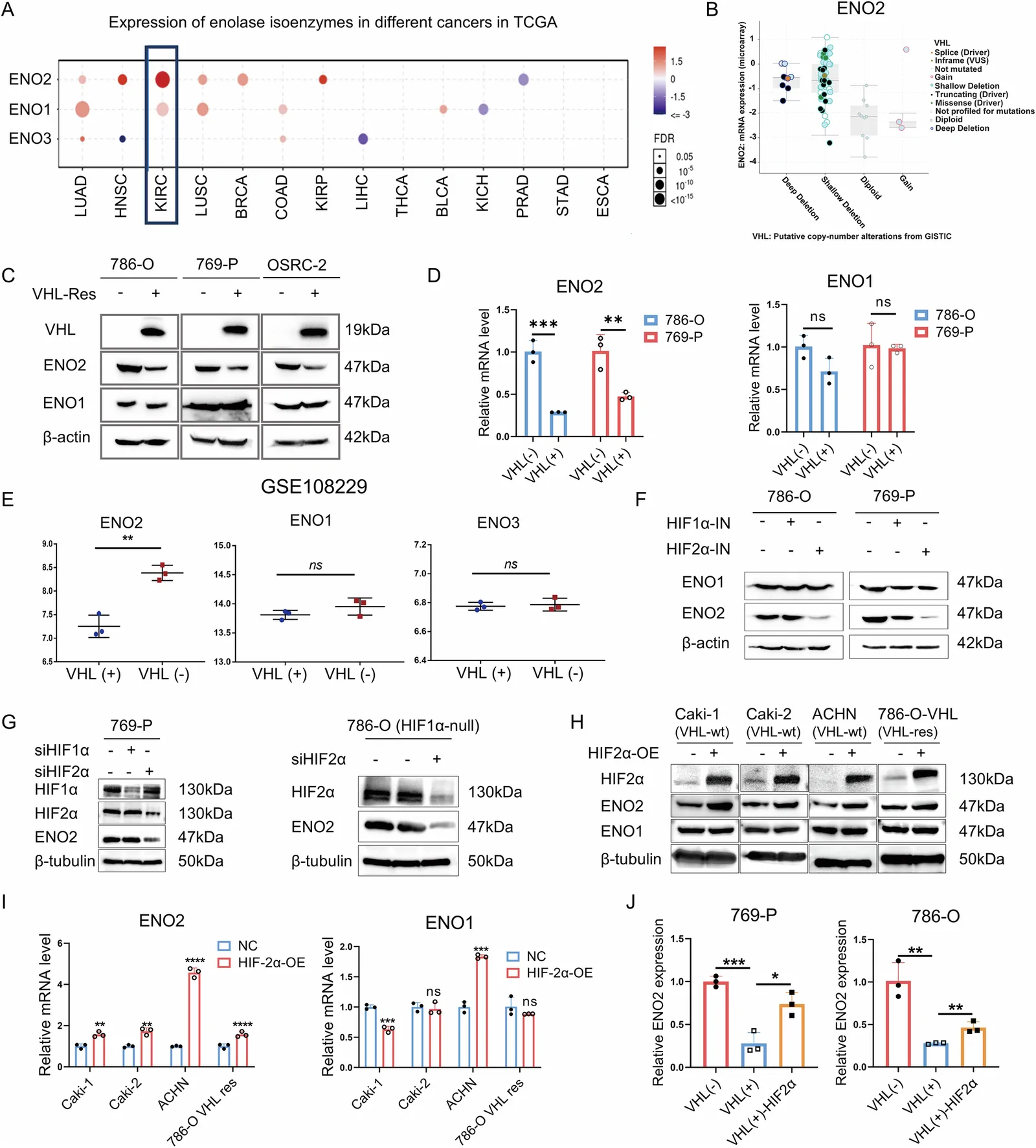
VHL loss drives ENO2 activation via HIF2α. **A** TCGA pan-cancer analysis of ENO1, ENO2, and ENO3 expression across various cancer types. **B** Box plot illustrating ENO2 mRNA expression (z-score) in TCGA KIRC tumor samples stratified by *VHL* copy-number status, as determined by GISTIC analysis using the cBioPortal platform (https://www.cbioportal.org). Each dot represents a single tumor sample, with color and outline indicating the mutation type (truncating, missense, inframe, splice, or not mutated) and copy-number category (Deep Deletion, Shallow Deletion, Diploid, or Gain), respectively. **C** Western blot analysis of ENO1 and ENO2 expression in VHL-deficient and VHL-rescued ccRCC cell lines. **D** RT-qPCR analysis of ENO1 and ENO2 expression in 786-O and 769-P cells with or without VHL reconstitution. **E** GSE108229 dataset analysis showing ENO1, ENO2, and ENO3 expression in 786-O cells with or without VHL reconstitution. **F** Western blot analysis of ENO1 and ENO2 in 786-O and 769-P cells treated with either the HIF1α inhibitor BAY87-2243 or the HIF2α inhibitor PT2385. **G** Western blot analysis showing the effects of HIF1α or HIF2α silencing on ENO2 expression in 769-P cells, and the effect of HIF2α silencing in HIF1α-null 786-O cells. **H** Western blot analysis validating HIF2α overexpression and its effect on ENO2 and ENO1 protein expression in ccRCC cell lines (Caki-1, Caki-2, ACHN, and VHL-rescued 786-O). **I** RT-qPCR assays showing the effect of HIF2α overexpression on ENO1 and ENO2 expression in ccRCC cell lines (Caki-1, Caki-2, ACHN, and VHL-rescued 786-O). **J** RT-qPCR analysis of ENO2 expression in parental VHL-deficient and VHL-rescued 786-O and 769-P cells, with or without HIF2α overexpression in the VHL-rescued background. Data are presented as mean ± SD. Statistical analyses were performed using unpaired two-tailed Student’s *t* tests or one-way ANOVA, as appropriate. *p < 0.05, **p < 0.01, ***p < 0.001, ****p < 0.0001; ns not significant.

TCGA pan-cancer analysis reveals that ENO2 upregulation shows obviously heightened prominence in ccRCC (Fig. 2A), strongly correlating with *VHL* mutations and copy number alterations (Fig. 2B), which are key drivers of glycolytic addiction in this malignancy [1, 7, 8]. These findings suggest that ENO2 activation in ccRCC is likely driven by VHL loss.

Restoration of wild-type VHL in VHL-deficient ccRCC cell lines significantly suppressed ENO2 expression at both protein and mRNA levels, while leaving ENO1 expression unaffected (Fig. 2C, D). This selective repression was corroborated by transcriptome analysis (GSE108229) from an independent study [25], which demonstrated that VHL loss specifically induces ENO2 activation without altering ENO1 or ENO3 expression (Fig. 2E). Collectively, these data confirm that VHL deficiency drives selective ENO2 upregulation in ccRCC.

To delineate the specific HIF-α isoform mediating this effect, we treated VHL-null ccRCC cells with HIF1α- and HIF2α-specific inhibitors. Notably, only HIF2α inhibition suppressed ENO2 expression (Fig. 2F). This finding was validated by siRNA-mediated silencing, which showed that knockdown of HIF2α, but not HIF1α, markedly reduced ENO2 expression (Fig. 2G). We subsequently overexpressed HIF2α in ccRCC cells (Caki-1, Caki-2, ACHN, and VHL-rescued 786-O cell lines) with low endogenous HIF2α and found that HIF2α preferentially enhanced ENO2 expression (Fig. 2H–I). Crucially, HIF2α overexpression significantly restored ENO2 expression in VHL-rescued ccRCC cells (Fig. 2J). Furthermore, analysis of a public HIF2α ChIP-seq dataset from 786-O cells (GSE34871) revealed a distinct binding peak within the ENO2 promoter region (Fig. S2). These data establish HIF2α as the dominant mediator of ENO2 activation following VHL loss.

### ENO2 mediates VHL deficiency-driven glycolytic reprogramming and malignant progression in ccRCC

Consistent with prior reports [5, 7], we demonstrated that VHL loss serves as a pivotal regulator of glycolytic addiction (Fig. S3A) and multifaceted malignant phenotypes (Fig. S3B–E) in ccRCC, promoting uncontrolled proliferation (Fig. S3B), apoptosis evasion (Fig. S3C), invasive dissemination (Fig. S3D), and angiogenesis (Fig. S3E). Importantly, these oncogenic effects depended heavily on glycolysis (Fig. S3B–E).

**Fig. 3:**
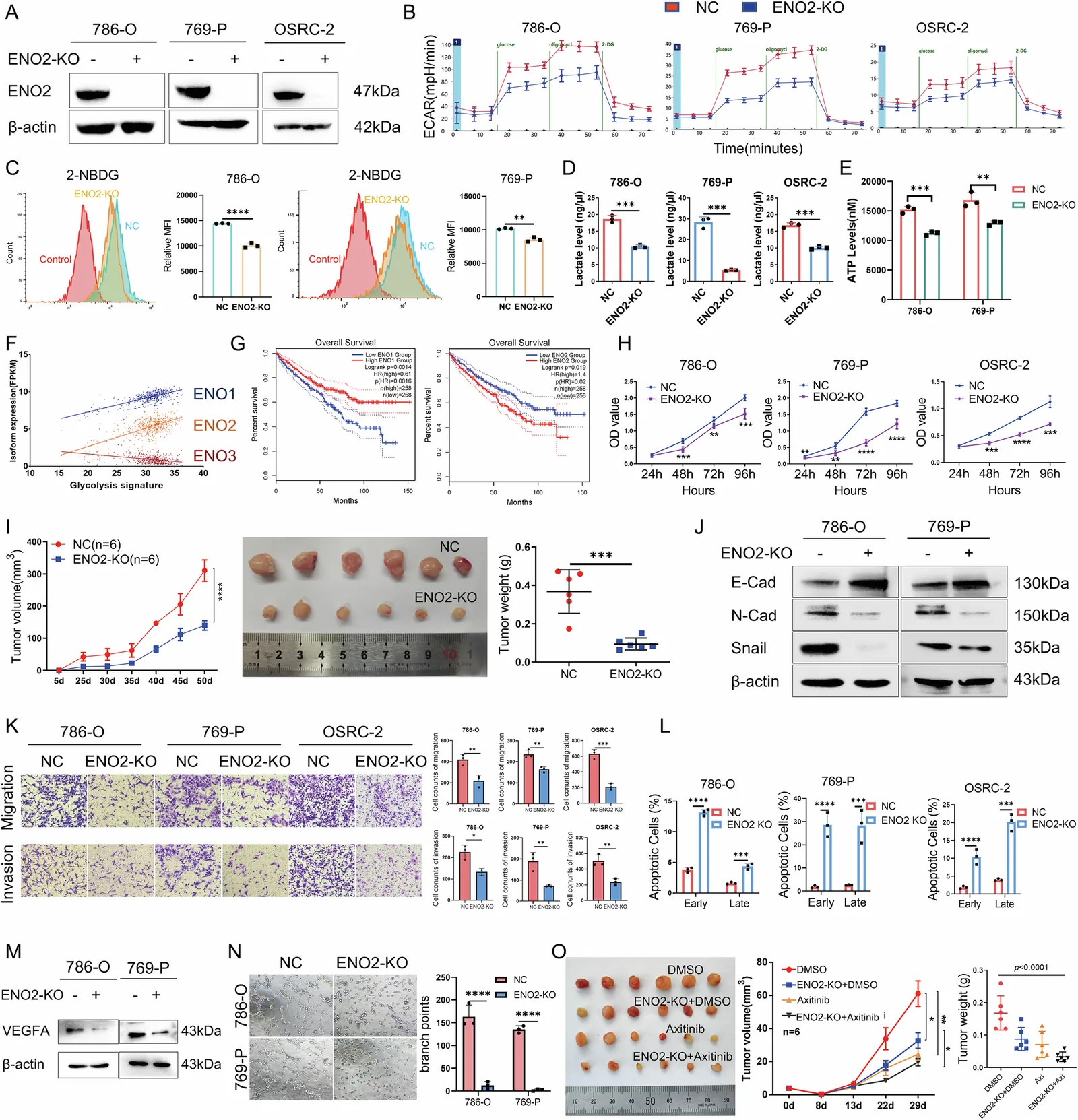
ENO2 mediates VHL deficiency-driven glycolytic reprogramming and malignant progression in ccRCC. **A** Western blot confirmation of ENO2 knockout (ENO2-KO) in VHL-deficient 786-O, 769-P, and OSRC-2 cells. **B** ECAR assessment of the effect of ENO2 ablation on glycolytic activity in three VHL-deficient ccRCC cell lines. **C** Glucose uptake analysis by flow cytometry measurement of 2-NBDG in VHL-deficient 786-O and 769-P cells with or without ENO2 ablation. **D** Quantification of extracellular lactate concentrations in ENO2-ablated and control 786-O, 769-P, and OSRC-2 cells. **E** Measurement of intracellular ATP levels in ENO2-ablated and control 786-O and 769-P cells. **F** TCGA analysis showing the correlation of ENO1, ENO2, or ENO3 expression with the glycolysis signature (defined by five key glycolytic enzymes: SLC2A1, HK2, PFKL, PKM, and LDHA) in the KIRC cohort. **G** Kaplan–Meier survival analysis showing the association of ENO1 or ENO2 expression with patient overall survival in the TCGA KIRC cohort using GEPIA2 online tools. **H** CCK-8 assessment of cell proliferation in ENO2-ablated and control 786-O, 769-P, and OSRC-2 cells. **I** Tumor growth curves and tumor weight of subcutaneous xenografts derived from ENO2-ablated and control 786-O cells in nude mice. **J** Western blot analysis of EMT markers in ENO2-ablated or control 786-O and 769-P cells. **K** Transwell assays evaluating cell migration and invasion in ENO2-ablated and control 786-O, 769-P, and OSRC-2 cells. **L** Flow cytometry analysis of early and late apoptosis in ENO2-ablated and control 786-O, 769-P, and OSRC-2 cells. **M** Western blot analysis of VEGFA expression in ENO2-ablated or control 786-O and 769-P cells. **N** Endothelial tube formation assays using conditioned medium from ENO2-ablated or control 786-O and 769-P cells. **O** Tumor volume, growth curve and tumor weight assays to evaluate the effect of ENO2 ablation combined with or without axitinib treatment (5 mg/kg) on the growth of subcutaneous xenografts derived from VHL-deficient 786-O cells. Data are presented as mean ± SD. Statistical analyses were performed using unpaired two-tailed Student’s *t* tests or one-way ANOVA, as appropriate. *p < 0.05, **p < 0.01, *** *p* < 0.001, ****p < 0.0001; ns not significant.

To investigate whether the physiologically redundant enolase isoform ENO2 contributes to VHL deficiency-induced metabolic dysregulation, we performed Extracellular Acidification Rate (ECAR) profiling. Notably, genetic ablation of ENO2 significantly attenuated glycolytic capacity across the three VHL-deficient ccRCC cell lines—786-O, 769-P, OSRC-2 (Fig. 3A, B). This was further supported by significantly reductions in glucose uptake (validated by 2-NBDG assays) (Fig. 3C), lactate secretion (Fig. 3D), and ATP generation (Fig. 3E).

TCGA analysis indicates that both ENO2 and ENO1 (but not ENO3) are associate with glycolysis signature in ccRCC (Fig. 3F). Given the distinct expression patterns of ENO1 and ENO2 under physiological versus pathological conditions, we propose that ENO1 sustains baseline glycolytic homeostasis (comparable to normal) in ccRCC, whereas ENO2 mediates the oncogenic glycolytic flux triggered by VHL loss. Survival analysis showed that ENO1 expression correlates with favorable prognosis, while ENO2 predicts poor prognosis in ccRCC (Fig. 3G).

Functional investigations demonstrated that ENO2 ablation comprehensively suppressed malignant behaviors in VHL-deficient ccRCC models, as evidenced by impaired proliferation in vitro (Fig. 3H), suppressed subcutaneous and orthotopic tumor growth in vivo (Fig. 3I; Fig. S4A–B), inactivated epithelial-mesenchymal transition (EMT) (Fig. 3J), attenuated invasive potential (Fig. 3K), decreased apoptosis evasion (Fig. 3L; Fig. S4C), as well as reduced VEGFA production (Fig. 3M; Fig. S4D) and angiogenesis (Fig. 3N). By contrast, ENO2 ablation in VHL-rescued 786-O and VHL-wild-type Caki-1 cells showed no appreciable effects on cell proliferation or migration (Fig. S4E–F), supporting a VHL loss-specific dependency on ENO2. ENO2 ablation also improved the therapeutic efficacy of the anti-angiogenic drug axitinib (Fig. 3O), and enhanced chemo-sensitivity to doxorubicin (Fig. S4G) and gemcitabine (Fig. S4H). Crucially, inhibiting glycolysis with 2-DG significantly diminished ENO2-induced malignancy (Fig. S5A–E), indicating that glycolytic activity underpins ENO2-mediated oncogenesis in VHL-deficient ccRCC.

### Targeting ENO2 specifically suppresses glycolysis in VHL-deficient ccRCC

**Fig. 4:**
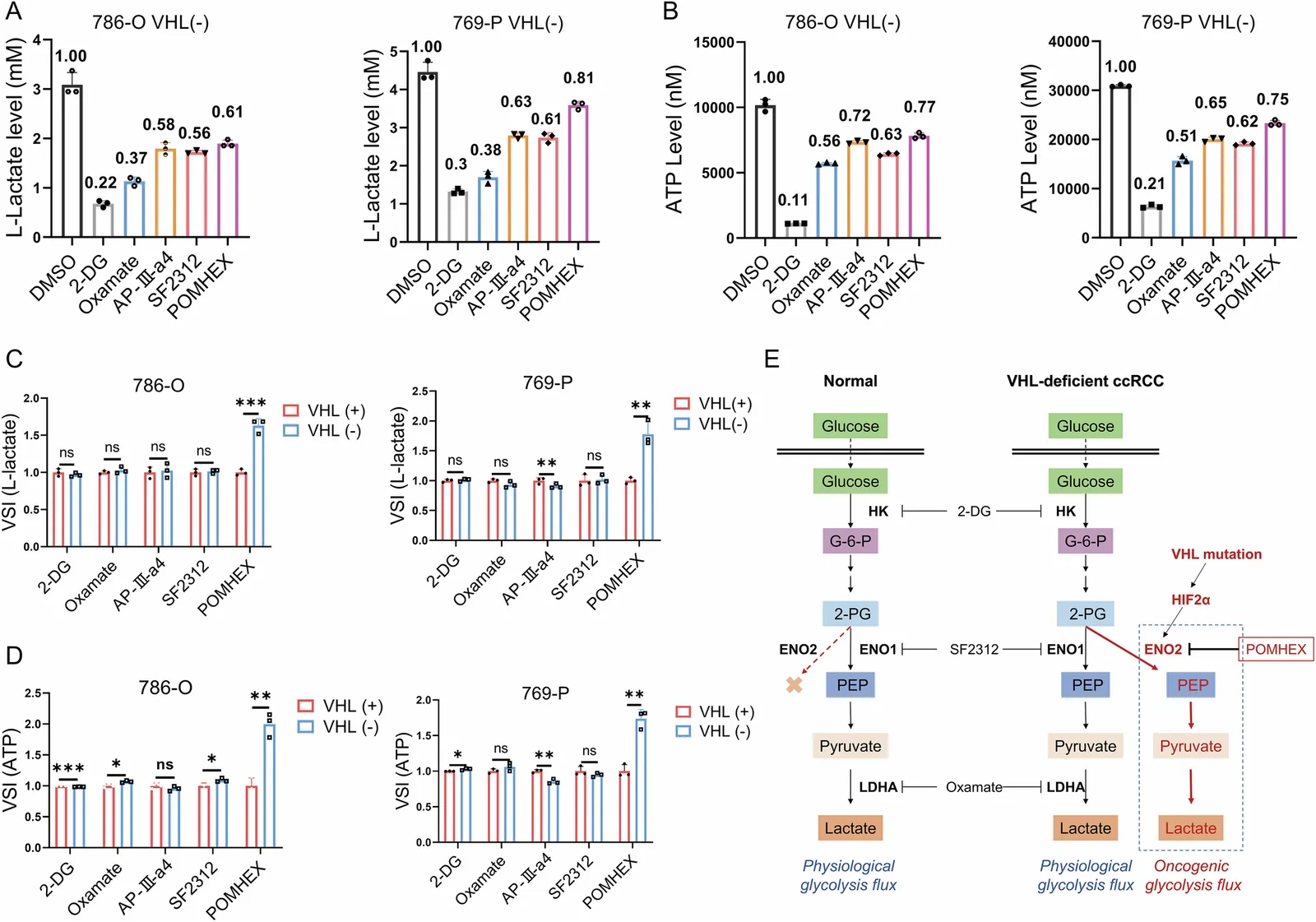
Targeting ENO2 specifically suppresses glycolysis in VHL-deficient ccRCC. **A** L-lactate measurements in VHL-deficient 786-O and 769-P ccRCC cells treated with glycolysis inhibitors (2-DG, oxamate, AP-III-a4, SF2312, and POMHEX). Fold changes (glycolysis inhibitor / DMSO) were shown. **B** Intracellular ATP measurements in VHL-deficient 786-O and 769-P cells treated with the indicated inhibitors. Fold changes (glycolysis inhibitor / DMSO) were shown. **C** VHL-loss selectivity index (VSI) measurements based on L-lactate analysis in 786-O and 769-P cells with or without VHL reconstitution under the indicated treatments. **D** VSI measurements based on ATP analysis in 786-O and 769-P cells with or without VHL reconstitution under the indicated treatments. **E** Schematic illustration of the distinct physiological and oncogenic glycolytic flux patterns in normal cells and VHL-deficient ccRCC cells, and the differential effects of conventional glycolysis inhibitors versus POMHEX. Data are presented as mean ± SD. Statistical analyses were performed using unpaired two-tailed Student’s *t* tests or one-way ANOVA, as appropriate. *p < 0.05, **p < 0.01, ***p < 0.001, ****p < 0.0001; ns not significant.

We next compared the efficacy and selectivity of the ENO2-specific inhibitor POMHEX with conventional glycolytic inhibitors (including hexokinase inhibitor 2-DG, LDHA inhibitor oxamate, and ENO1-preferential enolase inhibitors AP-III-a4/SF2312) in ccRCC models. Quantitative metabolic analyses of lactate secretion (Fig. 4A) and ATP production (Fig. 4B) confirmed that all inhibitors significantly suppressed glycolysis in VHL-deficient ccRCC cells (786-O and 769-P). However, POMHEX exhibited relatively weaker glycolytic inhibition compared to traditional inhibitors (Fig. 4A, B).

Notably, despite slightly weaker glycolytic inhibition potency, both lactate (Fig. 4C) and ATP quantification (Fig. 4D) analyses revealed that POMHEX exhibited remarkably higher selectivity for VHL (−) cells versus VHL (+) cells. This was evidenced by a VHL-loss selectivity index (VSI) of 1.6 to 2.0, substantially exceeding the negligible selectivity (VSI consistently <1.1) of conventional inhibitors (Fig. 4C, D).

Critically, POMHEX exerted minimal inhibitory effects on glycolysis in normal human renal proximal tubular epithelial cells (HK-2), as shown by marginally reduced lactate or ATP levels (Fig. S6). In contrast, all traditional inhibitors substantially compromised glycolysis in HK-2 cells (Fig. S6).

These results indicate that while ENO2-targeted inhibition exhibits weaker inhibitory efficacy, it demonstrates markedly stronger specificity for ccRCC with VHL deficiency (which constitutes >90% of all ccRCC cases) and causes significantly less interference with glycolysis in normal cells. This provides a critical therapeutic advantage over conventional inhibitors (Fig. 4E).

### Targeting ENO2 broadly suppresses VHL deficiency-activated oncogenes through lactate and interferon signaling

**Fig. 5:**
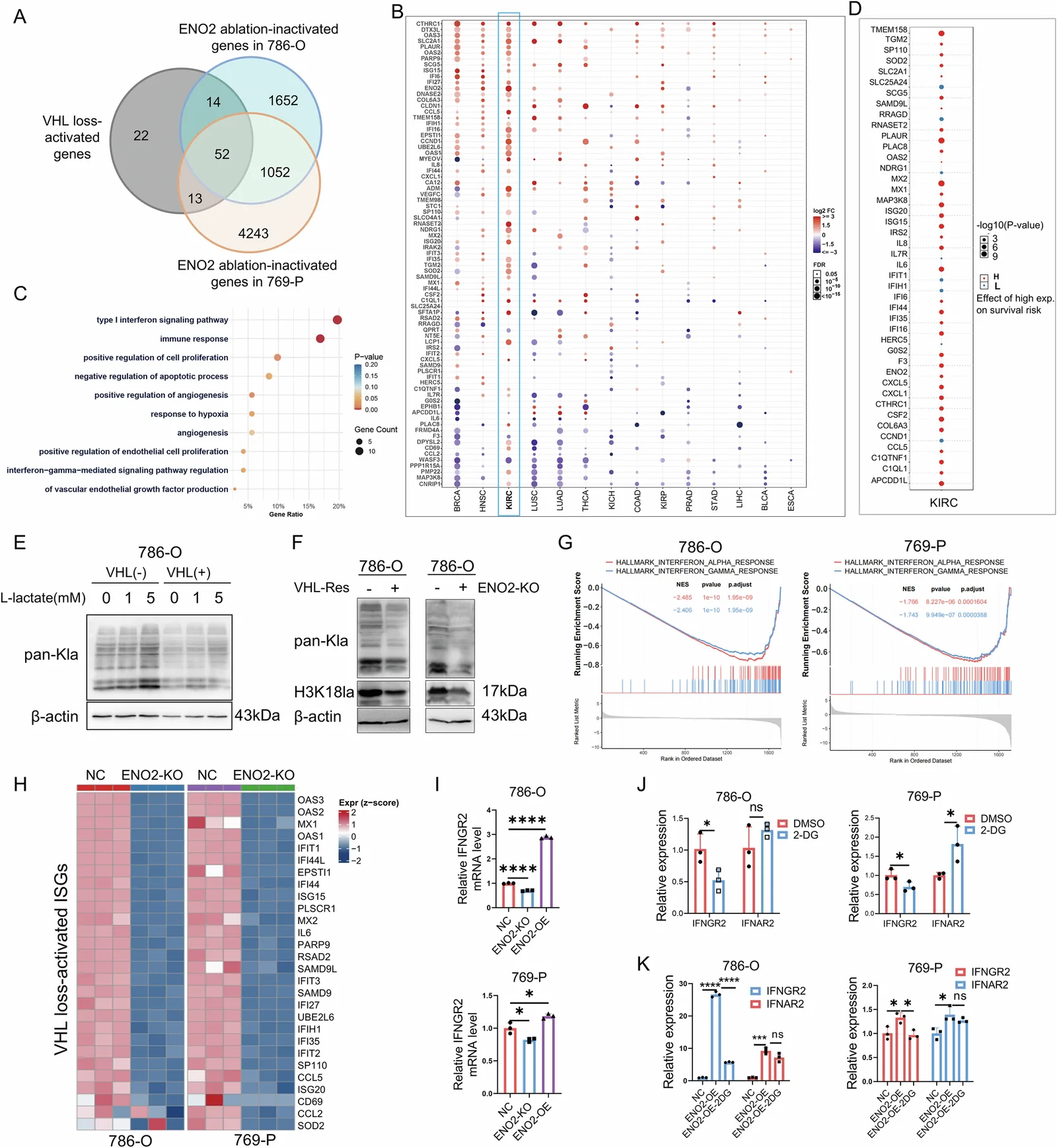
Targeting ENO2 broadly suppresses VHL deficiency-activated oncogenes through lactate and interferon signaling. **A** Venn diagram showing the overlap of VHL loss-activated genes (top 101 in GSE108229) and ENO2 ablation-inactivated genes in VHL-deficient 786-O and 769-P cells. **B** Expression patterns of the overlapping genes across TCGA pan-cancer datasets. **C** Gene ontology (GO) analysis of enriched biological processes among the overlapping genes. **D** Survival association of the overlapping genes in the TCGA KIRC cohort. **E** Western blot analysis of pan-lysine lactylation (pan-Kla) in 786-O cells treated with increasing concentrations of L-lactate under VHL (+) or VHL (−) background. **F** Western blot assessment of the effects of VHL reconstitution and ENO2 knockout on pan-Kla and H3K18la in 786-O cells. **G** GSEA plots showing the enrichment of ENO2-regulated genes in interferon-alpha and interferon-gamma response pathways in 786-O and 769-P cells. **H** Heatmap showing VHL loss-activated interferon-stimulated genes (ISGs) that are suppressed by ENO2 ablation in 786-O and 769-P cells. **I** RT-qPCR analysis of IFNGR2 mRNA expression in control (NC), ENO2-knockout (KO), and ENO2-overexpressing (OE) 786-O and 769-P cells. **J** RT-qPCR analyses showing the effect of 2-DG (5 mM, 48 h) on endogenous IFNGR2 and IFNAR2 expression in 786-O and 769-P cells. **K** RT-qPCR analyses showing the effect of 2-DG-mediated glycolysis inhibition on ENO2-induced IFNGR2 and IFNAR2 expression in 786-O and 769-P cells. Data are presented as mean ± SD. Statistical analyses were performed using unpaired two-tailed Student’s *t* tests or one-way ANOVA, as appropriate. *p < 0.05, **p < 0.01, ***p < 0.001, ****p < 0.0001; ns not significant.

To elucidate the molecular mechanisms underlying ENO2-mediated inhibition of malignant phenotypes in VHL-deficient ccRCC, we performed genome-wide transcriptomic profiling. Comparative analysis (Fig. 5A) demonstrated that ENO2 ablation effectively reversed 78.2% (79/101) of VHL loss-activated downstream targets [25]. Critically, these genes were predominantly upregulated in ccRCC (Fig. 5B), functionally associated with critical oncogenic processes including cell proliferation, angiogenesis, and apoptosis resistance (Fig. 5C), and primarily correlated with poor prognosis (Fig. 5D). These data suggest ENO2 as a critical regulator of VHL deficiency-driven transcriptional reprogramming in ccRCC tumorigenesis.

To further elucidate the mechanistic basis for ENO2’s broad regulation of VHL targets, we first focused on lactate, the terminal glycolytic metabolite known to regulate histone lactylation and global gene expression [26]. We confirmed that lactate induces protein lactylation in ccRCC (Fig. 5E), and VHL deficiency markedly enhances both pan-lactylation and specific H3K18 lactylation (Fig. 5E, F). Notably, ENO2 depletion phenocopies the lactylation suppression observed upon VHL reconstitution (Fig. 5F).

Additionally, transcriptomic analysis revealed significant enrichment of ENO2-regulated genes in interferon signaling pathways in both 786-O and 769-P cells (Fig. 5G). Importantly, VHL loss-activated targets included a number of interferon-stimulated genes (ISGs) that were potently suppressed by ENO2 ablation in both cell lines (Fig. 5H). Subsequent validation of representative ISGs, including OAS1, OAS2, ISG15, MX1, MX2, and IFIT1, further demonstrated that ENO2 is required for the VHL loss-driven activation of interferon signaling (Fig. S7A). Moreover, 2-DG-mediated glycolytic inhibition phenocopied ENO2 ablation in suppressing these representative ISGs (Fig. S7B). RNA-seq data further revealed that ENO2 may regulate interferon signaling by activating interferon receptors (Fig. S8). Validation showed that ENO2 knockout significantly downregulated IFNGR2 expression, while ENO2 overexpression upregulated it (Fig. 5I). Remarkably, IFNGR2 reconstitution in ENO2-deficient 786-O and 769-P ccRCC cells restored the responsiveness of ISGs (OAS1, OAS2, ISG15, MX1, MX2, and IFIT1) to IFNγ (Fig. S7C). Glycolytic inhibition with 2-DG selectively reduced IFNGR2 (interferon γ receptor) expression but did not affect IFNAR2 (interferon α and β receptor) expression (Fig. 5J). Moreover, 2-DG-mediated glycolytic inhibition abrogated ENO2-induced IFNGR2 upregulation without affecting IFNAR2 upregulation (Fig. 5K). These findings suggest that ENO2-maintained glycolytic flux may also mediate the VHL deficiency-driven oncogenic transcriptional program by the IFNGR2-interferon axis.

### MDK exemplifies a critical effector downstream of the VHL-HIF2α-ENO2-glycolysis oncogenic axis

**Fig. 6:**
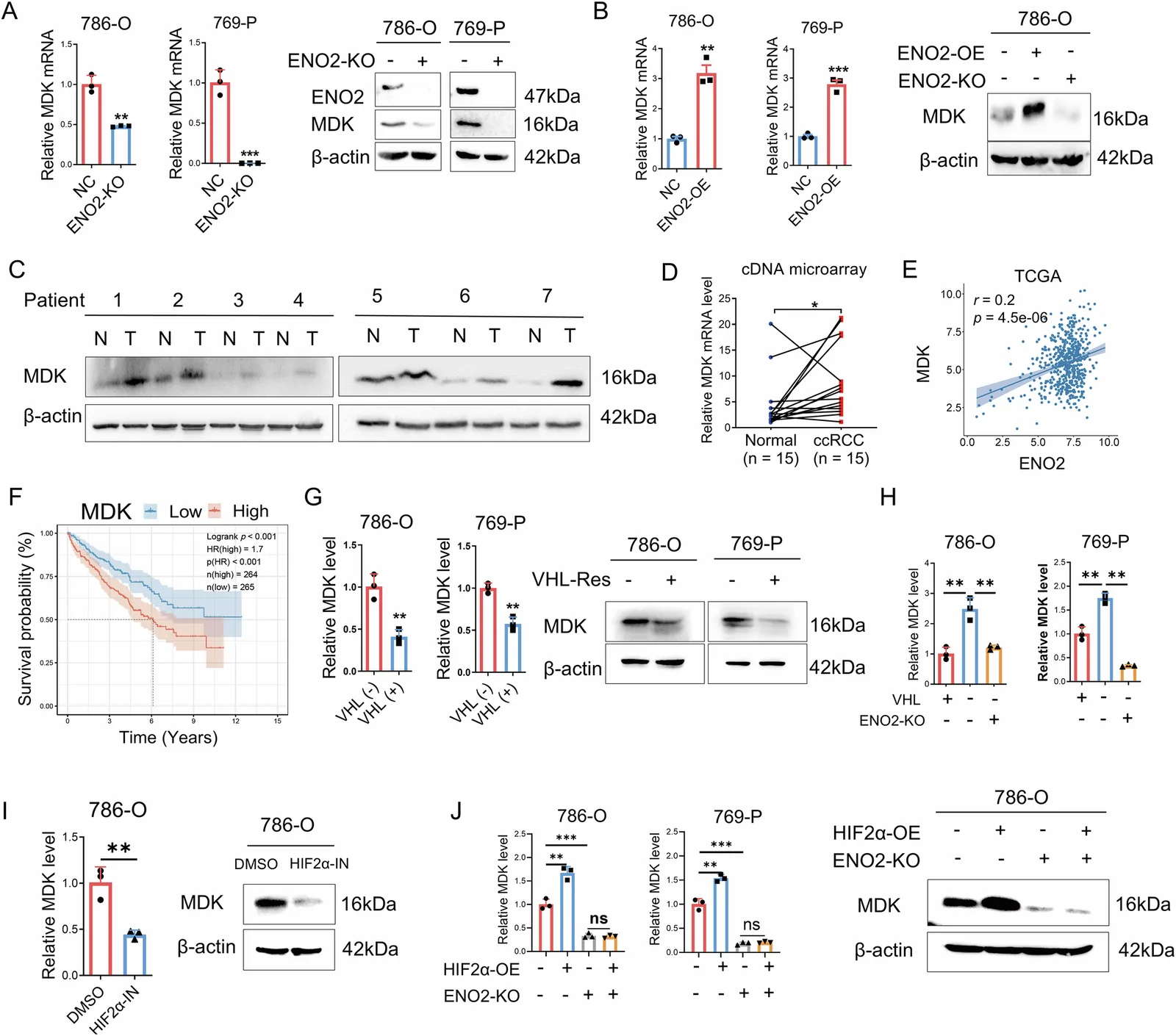
MDK is a critical downstream target of the VHL-HIF2α-ENO2 axis in ccRCC. **A** RT-qPCR and western blot analyses showing the effect of ENO2 ablation on MDK expression in 786-O and 769-P cells. **B** RT-qPCR and western blot analyses showing the effect of ENO2 overexpression on MDK expression in 786-O and 769-P cells. **C** Western blot analysis showing upregulation of MDK protein in paired tumor (T) compared to adjacent normal (N) tissues from 7 ccRCC patients. **D** Assessment of MDK mRNA expression in 15 paired samples in a ccRCC cDNA microarray. **E** Correlation between ENO2 and MDK expression in ccRCC samples from the TCGA KIRC dataset. **F** Kaplan–Meier survival analysis of overall survival in TCGA ccRCC patients stratified by MDK expression levels. **G** RT-qPCR and western blot analyses showing the effect of VHL reconstitution on MDK expression in VHL-deficient 786-O and 769-P cells. **H** RT-qPCR analyses showing the effect of ENO2 ablation on VHL loss-induced MDK expression in 786-O and 769-P cells. **I** RT-qPCR and western blot analyses showing the effect of HIF2α inhibitor (PT2385) on MDK expression in 786-O cells. **J** RT-qPCR and western blot analyses showing the effect of ENO2 ablation on HIF2α overexpression (OE)-induced MDK expression in 786-O and 769-P cells. Data are presented as mean ± SD. Statistical analyses were performed using unpaired two-tailed Student’s *t* tests or one-way ANOVA, as appropriate. * *p* < 0.05, **p < 0.01, ***p < 0.001, ****p < 0.0001; ns not significant.

Our RNA-seq analysis revealed midkine (MDK), a well-known pleiotropic oncoprotein, as a common downstream target of ENO2 in 786-O and 769-P cells (Fig. S9). This was subsequently validated at both mRNA and protein levels, demonstrating that ENO2 ablation suppressed and ENO2 overexpression enhanced MDK expression in both ccRCC cell lines (Fig. 6A, B). Clinically, MDK is significantly upregulated in ccRCC specimens (Fig. 6C, D), positively correlated with ENO2 expression (Fig. 6E), and indicates poor prognosis (Fig. 6F).

Moreover, VHL reconstitution in both VHL-null ccRCC cell lines (786-O and 769-P) significantly reduced MDK expression (Fig. 6G), while ENO2 ablation effectively abrogated VHL deficiency-induced MDK upregulation (Fig. 6H). Parallel experiments with HIF2α modulation yielded consistent results. HIF2α inhibitor treatment significantly downregulated (Fig. 6I), while HIF2α overexpression upregulated MDK expression in ccRCC cells (Fig. 6J), with ENO2 ablation again abolishing HIF2α-driven MDK expression (Fig. 6J). These results collectively identify a VHL-HIF2α-ENO2-MDK signaling cascade.

**Fig. 7:**
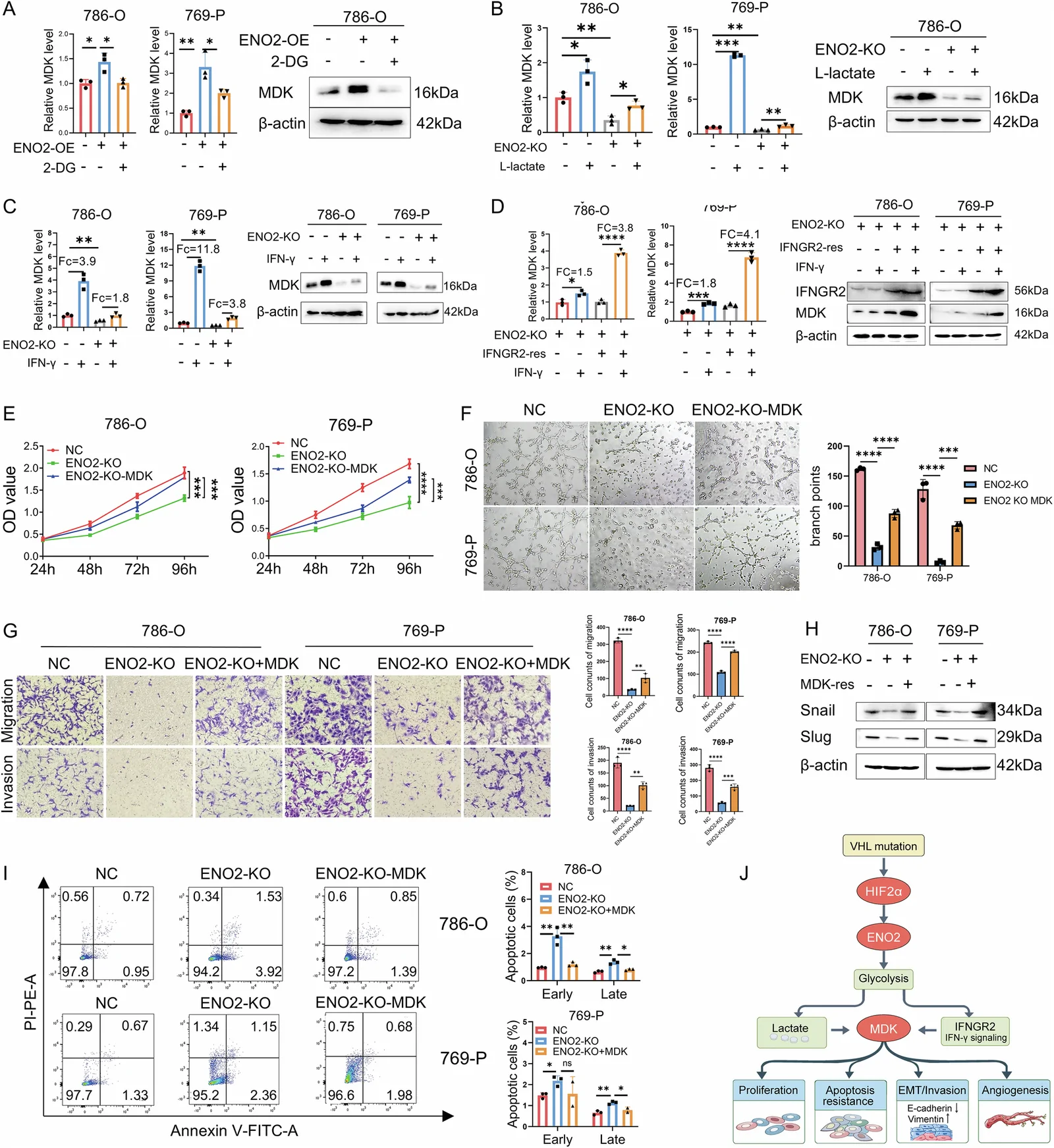
MDK reconstitution partially rescues ENO2 depletion-induced tumor suppression phenotypes in ccRCC. **A** RT-qPCR and western blot assays showing the effect of 2-DG-mediated glycolysis inhibition on ENO2 overexpression-induced MDK expression in 786-O and 769-P cells. **B** RT-qPCR and western blot assays showing the effect of L-lactate (5 mM, 24 h) on MDK expression in control and ENO2-deleted 786-O and 769-P cells. **C** RT-qPCR and western blot assays showing the effect of IFN-γ (10 ng/mL, 24 h) on MDK expression, and the impact of ENO2 ablation on IFN-γ-induced MDK expression in 786-O and 769-P cells. **D** RT-qPCR and western blot assays showing the effect of IFNGR2 reconstitution (res) on MDK responsiveness to IFN-γ in 786-O and 769-P cells. **E** CCK-8 assays evaluating the effect of MDK reconstitution on ENO2 ablation-induced cell proliferation suppression in 786-O and 769-P cells. **F** Endothelial tube formation assays evaluating the effect of MDK reconstitution on ENO2 ablation-induced angiogenesis suppression in 786-O and 769-P cells. **G** Transwell assays assessing the effect of MDK reconstitution on ENO2 ablation-induced migration and invasion suppression in 786-O and 769-P cells. **H** Western blot assays examining the effect of MDK reconstitution (MDK-res) on ENO2 ablation-induced mesenchymal signature suppression. **I** Flow cytometry assays with Annexin V/PI staining to determine the effect of MDK reconstitution on ENO2 ablation-induced apoptosis in 786-O and 769-P cells. **J** Schematic model of the mechanism by which the VHL–HIF2α–ENO2 axis promotes ccRCC progression. Data are presented as mean ± SD. Statistical analyses were performed using unpaired two-tailed Student’s *t* tests or one-way ANOVA, as appropriate.* *p* < 0.05, **p < 0.01, ***p < 0.001, ****p < 0.0001; ns not significant.

Further investigation revealed that glycolysis inhibition with 2-DG substantially blocked ENO2-mediated MDK activation (Fig. 7A), suggesting glycolytic flux as a metabolic regulator. Lactate, a key glycolytic metabolite, significantly upregulated MDK expression in ccRCC cells (Fig. 7B), and L-lactate supplementation partially rescued MDK expression in ENO2-knockout cells (Fig. 7B).

Moreover, building on our recent identification of MDK as a novel IFNγ-responsive gene target [27] and ENO2’s role in IFNGR2-interferon signaling, we hypothesized that ENO2 may also activate MDK via IFNGR2-interferon signaling. Validation at both mRNA and protein levels showed that IFNγ activates MDK expression, while ENO2 knockdown diminishes this stimulation (Fig. 7C). Notably, IFNGR2 reconstitution in ENO2-deficient ccRCC cells substantially restored MDK responsiveness to IFNγ (Fig. 7D), identifying IFNGR2-interferon signaling as an alternative mechanism for ENO2-dependent MDK regulation.

Functional investigations showed that MDK reconstitution broadly rescued the tumor-suppressive effects of ENO2 ablation in VHL-deficient ccRCC cells, significantly restoring proliferation (Fig. 7E), angiogenesis (Fig. 7F), invasion and migration (Fig. 7G), mesenchymal traits (Fig. 7H), and apoptosis resistance (Fig. 7I). These findings collectively confirm MDK as a key effector executing the oncogenic program of the VHL-HIF2α-ENO2-glycolysis cascade in ccRCC (Fig. 7J).

### Pharmacological targeting of ENO2 effectively eliminates VHL-deficient ccRCC while preserving VHL-intact cells

**Fig. 8:**
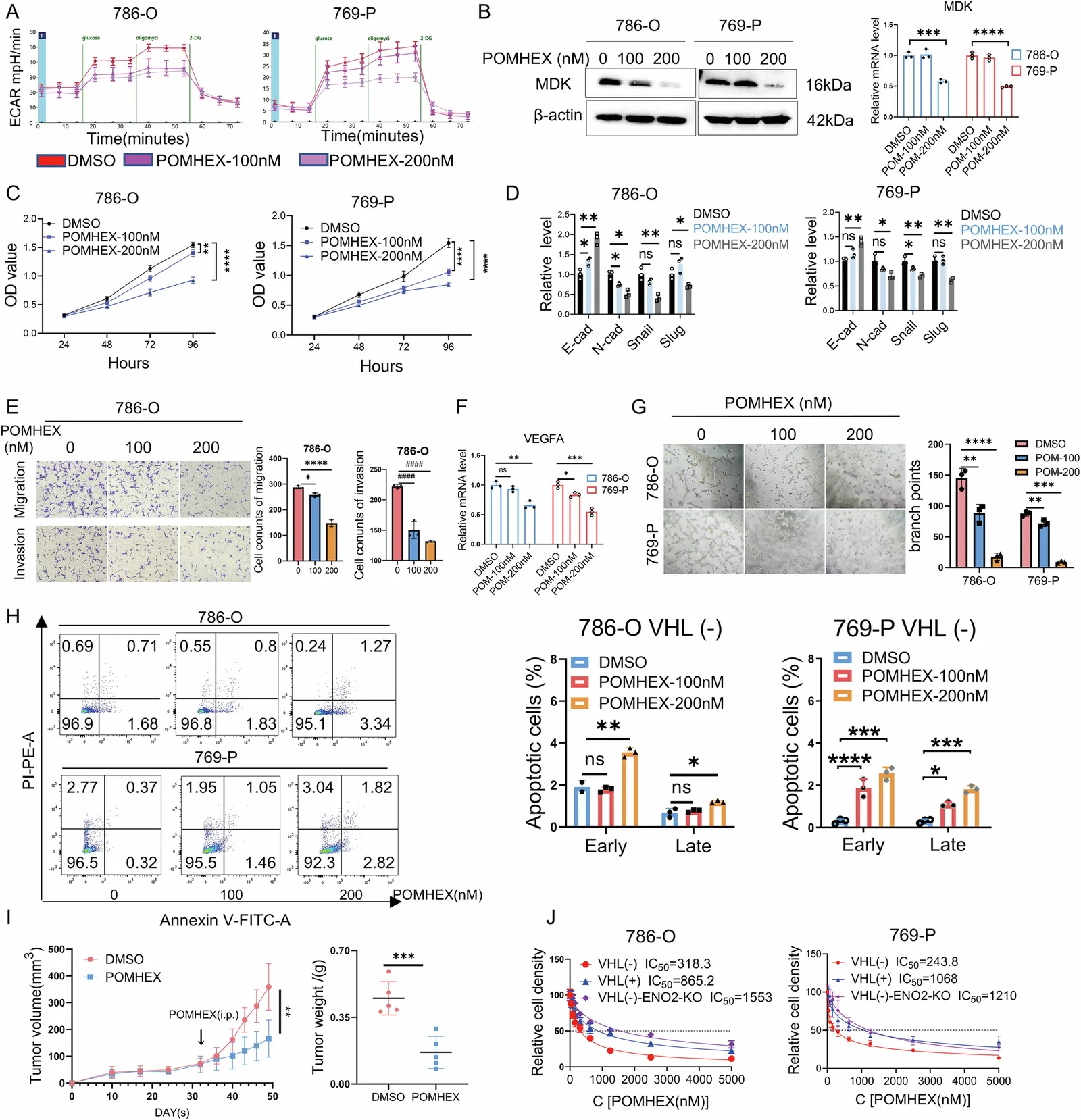
Pharmacological targeting of ENO2 effectively eliminates VHL-deficient ccRCC while preserving VHL-intact cells. **A** ECAR assays to determine the effect of POMHEX (100 nM, 200 nM) treatment on glycolytic capacity in VHL-deficient 786-O and 769-P cells. **B** Western blot and RT-qPCR assays to determine the effect of POMHEX (100 nM, 200 nM) treatment on MDK expression in 786-O and 769-P cells. **C** CCK-8 assays of cell growth curves to determine the effect of POMHEX (100 nM, 200 nM) treatment on cell proliferation in VHL-deficient 786-O and 769-P cells. **D** RT-qPCR assays to determine the effect POMHEX treatment on EMT markers in VHL-deficient 786-O and 769-P cells. **E** Transwell assays to evaluate the effect of POMHEX treatment on cell migration and invasion in VHL-deficient 786-O cells. **F** RT-qPCR assays to evaluate the effect POMHEX treatment on VEGFA expression in VHL-deficient 786-O and 769-P cells. **G** Endothelial tube formation assays using conditioned medium from 786-O and 769-P cells treated with indicated concentrations of POMHEX. **H** Flow cytometry analysis of apoptosis in 786-O and 769-P cells treated with DMSO or POMHEX. **I** Evaluation of POMHEX antitumor efficacy in 786-O subcutaneous xenograft models via tumor growth curves and weight analysis. **J** CCK-8 determination of the IC_50_ values of POMHEX in VHL-null (−), VHL-rescued (+), and ENO2-ablated (KO) VHL-null ccRCC cells (786-O and 769-P). Data are presented as mean ± SD. Statistical analyses were performed using unpaired two-tailed Student’s *t* tests or one-way ANOVA, as appropriate. *p < 0.05, **p < 0.01, ***p < 0.001, ****p < 0.0001; ns not significant.

We next evaluated the therapeutic value of pharmacological ENO2 inhibition by POMHEX in ccRCC. As expected, POMHEX treatment markedly attenuated glycolytic capacity (Fig. 8A) and suppressed MDK expression (Fig. 8B) in VHL-deficient ccRCC cell lines. Functional investigations revealed that POMHEX exerted broad inhibitory effects on oncogenic processes in these cells, including cell proliferation (Fig. 8C), EMT (Fig. 8D), migration and invasion (Fig. 8E), VEGFA expression (Fig. 8F), angiogenesis (Fig. 8G), and apoptosis resistance (Fig. 8H). POMHEX also significantly suppressed tumor growth in VHL-deficient 786-O xenografts in vivo (Fig. 8I), without inducing observable toxicity or abnormalities in mice. IC_50_ measurements were conducted to assess the specificity of POMHEX. The 786-O and 769-P ccRCC cell lines with inherent VHL deficiency exhibited notable sensitivity to POMHEX, with IC_50_ values of 318.3 nM and 243.8 nM respectively (Fig. 8J). Intriguingly, VHL-reconstituted counterparts of these cell lines demonstrated significantly higher IC_50_ values (865.2 nM and 1068 nM) (Fig. 8J), indicating substantially reduced drug sensitivity. Most remarkably, ENO2-depleted VHL-deficient cells, where ENO2 expression levels are comparable to normal cells and are not dispensable for cell survival, developed pronounced resistance to POMHEX (IC50: 1553 nM and 1210 nM) (Fig. 8J).

Similarly, ccRCC cells (Caki-1 and ACHN) with innate wild-type VHL expression displayed IC_50_ values (984.2 nM and 835.1 nM) comparable to VHL-rescued cells (Fig. S10). The neuroendocrine tumor line QGP showed even greater resistance (IC_50_: 2019 nM) (Fig. S10). Crucially, all six tested normal cell lines maintained exceptionally high IC_50_ values (ranging from 3000 to 7000 nM) (Fig. S10), demonstrating minimal drug sensitivity.

Collectively, these results establish that pharmacological ENO2 inhibition exhibits remarkable specificity for VHL-deficient ccRCC while maintaining excellent safety profiles in VHL-competent malignant and normal cells. This targeted approach may avoid the on-target normal toxicities associated with conventional glycolysis inhibitors in clinical applications.

## Discussion

Our study reveals that the *VHL*-mutant ccRCC selectively hijacks the normally redundant enolase isoenzyme ENO2 to fulfill its heightened glycolytic requirements. This finding enables the design of a targeted glycolysis inhibition strategy that selectively impairs VHL-deficient ccRCC cells while sparing VHL-wildtype normal and malignant cells.

Aerobic glycolysis (the Warburg effect), a hallmark of cancer metabolism, critically supports tumor growth by facilitating rapid ATP generation and biomass production [28–32]. While glycolytic inhibition represents a promising therapeutic strategy for ccRCC with prevalent *VHL* mutations, conventional approaches have been hampered by poor tumor specificity and systemic toxicity stemming from the essential housekeeping functions of their targeted glycolytic enzymes in normal metabolism [10–12].

Our work identifies ENO2 as an ideal metabolic target in ccRCC due to its unique expression pattern and functional properties. Emerging studies have also linked ENO2 to ccRCC progression, metabolic remodeling, and hypoxia-associated tumor adaptation [33–37]. Unlike the ubiquitously expressed ENO1 that maintains housekeeping glycolytic functions in normal tissues [19, 20], ENO2 exhibits minimal expression in most normal cells and demonstrates complete functional redundancy through compensatory ENO1 activity [19, 20, 23]. Notably, preclinical studies in non-human primates have demonstrated excellent tolerability of ENO2-specific inhibitors like POMHEX at therapeutic doses [22, 24], supporting the safety of this approach.

This functional redundancy extends to neural tissues, where ENO2 expression is relatively higher yet dispensable for cellular survival when ENO1 is present [22, 24]. This may be due to the modest glycolytic requirements and substantial ENO1 reserves in normal neural cells. Intriguingly, highly proliferative neural tumors display significantly reduced ENO2 expression compared to normal neural tissues, further corroborating ENO2’s dispensability under physiological conditions. Current literature confirms that ENO2 inhibition only proves cytotoxic in rare ENO1-null malignancies (e.g., certain glioblastomas) where ENO2 serves as the exclusive enolase activity source [22, 24].

The therapeutic potential of ENO2 inhibition lies in its context-dependent essentiality in cancer. While functionally redundant in normal physiology, ENO2 becomes critically required in VHL-deficient ccRCC owing to HIF2α-mediated upregulation and the amplified glycolytic demands. Our findings demonstrate that ENO2 ablation selectively impairs glycolytic flux and viability in VHL-deficient tumors while sparing VHL-intact normal and malignant cells, thereby creating a unique therapeutic window for developing ccRCC-specific metabolic intervention.

At the molecular level, ENO2 targeting disrupts VHL loss-driven oncogenic programs, particularly those involved in lactate and interferon signaling, including a pivotal effector MDK. This pleiotropic oncoprotein, extensively implicated in tumor progression, metastasis, and immune evasion, demonstrates strong associations with hypoxia across diverse malignancies [38–42].

Several limitations of the present study warrant acknowledgment to contextualize our findings and guide targeted future research. First, although multiple lines of evidence—including HIF2α inhibitor treatment, HIF2α genetic silencing, HIF2α overexpression, and HIF2α ChIP-seq data—consistently support HIF2α-dependent activation of ENO2, more direct validation of ENO2 promoter activity (e.g., targeted luciferase reporter assays with truncated/mutated promoter constructs, or site-directed mutagenesis of predicted HIF-binding elements) would further reinforce the specificity and robustness of this regulatory axis. Second, while our data demonstrate that ENO2-driven glycolytic flux shapes broad oncogenic transcriptional outputs through lactate-associated signaling and a glycolysis-dependent IFNGR2–interferon branch, the precise molecular underpinnings of these effects remain incompletely resolved. In particular, the causal role of lactylation in mediating ENO2-dependent transcriptional reprogramming, as well as the exact regulatory cascade governing ENO2-mediated control of IFNGR2 expression represent critical gaps that warrant further investigation.

The recognition of ENO2 as a *VHL* mutation-induced metabolic vulnerability in ccRCC establishes a paradigm for developing genotype-selective glycolytic therapies. By precisely targeting ENO2, these interventions can selectively disrupt the oncogenic glycolytic reprogramming that fuels ccRCC growth and progression, while minimizing toxicity to normal tissues. This precision medicine strategy aligns with contemporary efforts to develop more selective and less toxic cancer treatments. Future investigations should prioritize clinical translation of ENO2 inhibitors and exploration of synergistic combination regimens. Such targeted metabolic interventions offer considerable promise for improving therapeutic outcomes in ccRCC while maintaining excellent safety profiles.

Collectively, this study identifies the physiologically redundant enolase isoform ENO2 as a glycolytic vulnerability unique to ccRCC patients harboring VHL deficiency. This discovery enables the design of a precision glycolysis inhibition strategy that exhibits enhanced tumor selectivity while minimizing systemic toxicity. Furthermore, our work indicates that targeting physiologically redundant metabolic isoenzymes may serve as a promising precision medicine approach for cancers with specific genetic alterations.

## Supplementary information

supplement materials

WB raw data

## Data Availability

The RNA-seq data in this study were deposited at GEO under the accession number GSE303099.
