# Supplementary material for: Targeting enolase isoenzyme 2 enables precision glycolysis inhibition in VHL-mutant renal cell carcinoma
Source: Cell Death Dis. 2026 Jul 14;17(1):872. doi: 10.1038/s41419-026-09102-4 (PMC13645809; doi:10.1038/s41419-026-09102-4)
Supplement: Supplementary file 1 [file 41419_2026_9102_MOESM1_ESM.pdf]

Supplementary materials: Figure S1-S10; Table S1-S2

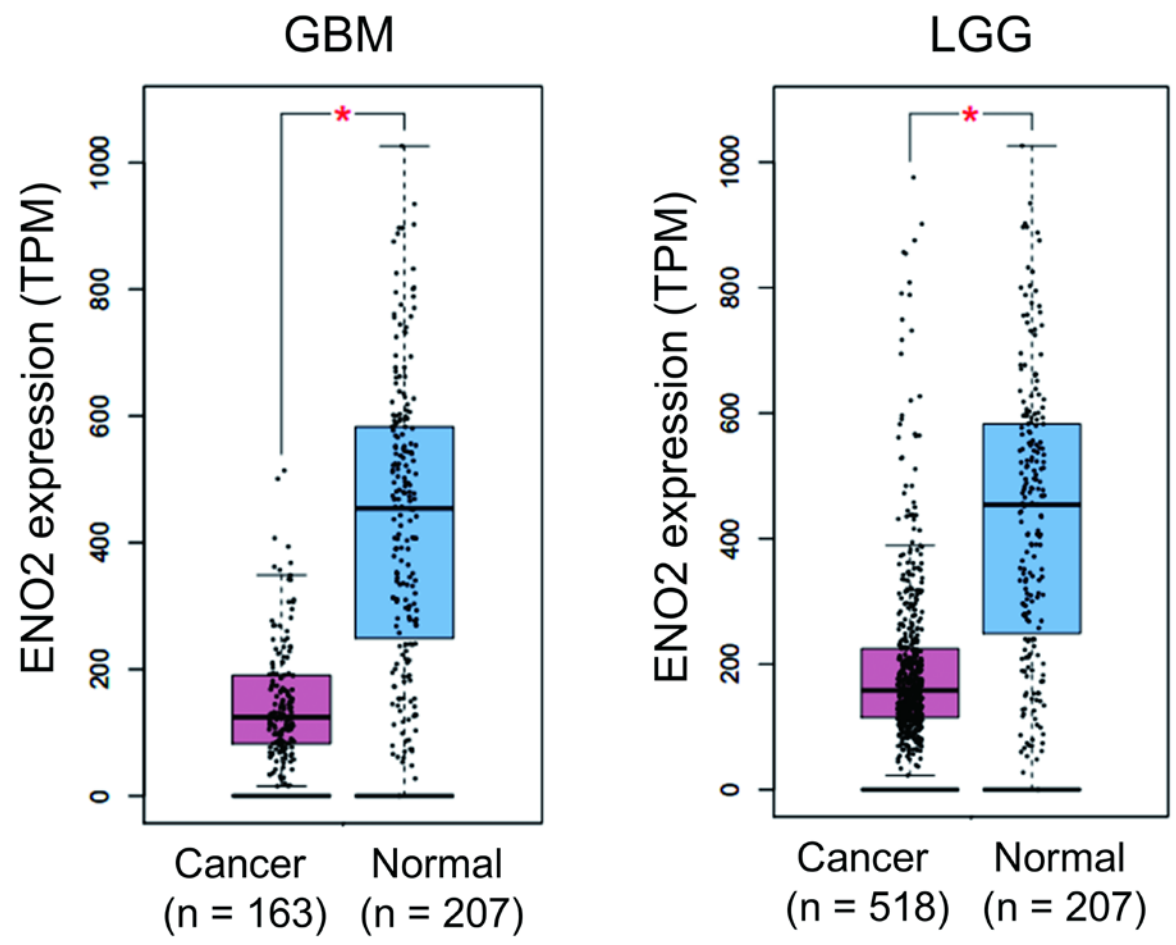

Figure S1. ENO2 is downregulated in neurological tumors compared to normal brain. GEPIA2 (http://gepia2.cancer-pku.cn/) analysis of ENO2 expression in glioblastoma multiforme (GBM) and brain lower grade glioma (LGG) tissues compared to normal brain tissues, based on TCGA and GTEx data. Box plots show the 25th, 50th, and 75th percentiles. Data were analyzed by unpaired two-tailed Student's t-test. $*p < 0.05$.

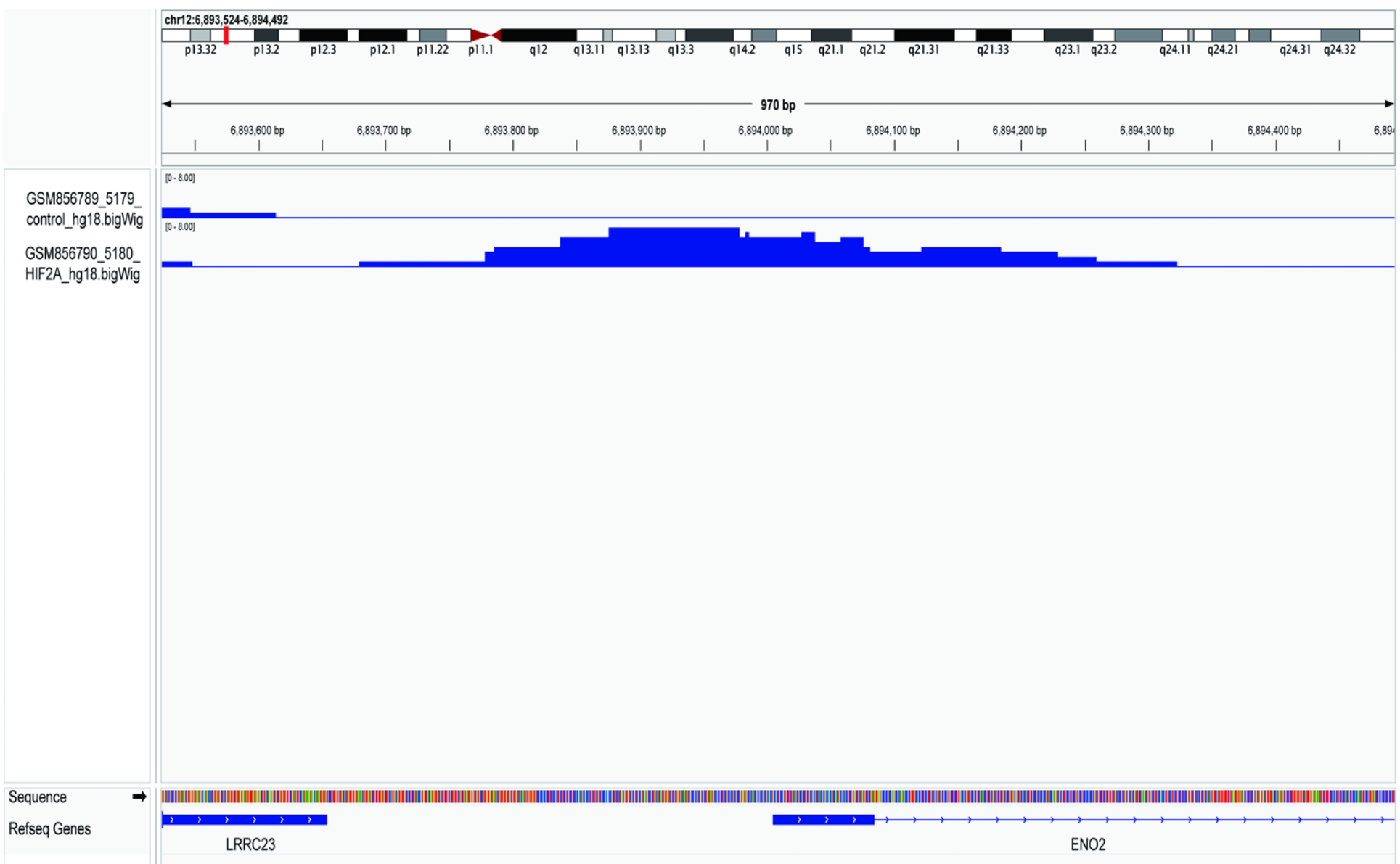

Figure S2. Public ChIP-seq data demonstrating HIF2α occupancy at the ENO2

promoter. IGV visualization of the ENO2 locus (hg18) reveals HIF2α enrichment, whereas the control track exhibits minimal signal. The displayed genomic region is chr12:6,893,524–6,894,492. RefSeq gene annotation is shown at the bottom. Public ChIP-seq data were obtained from the GEO database (GSE34871).

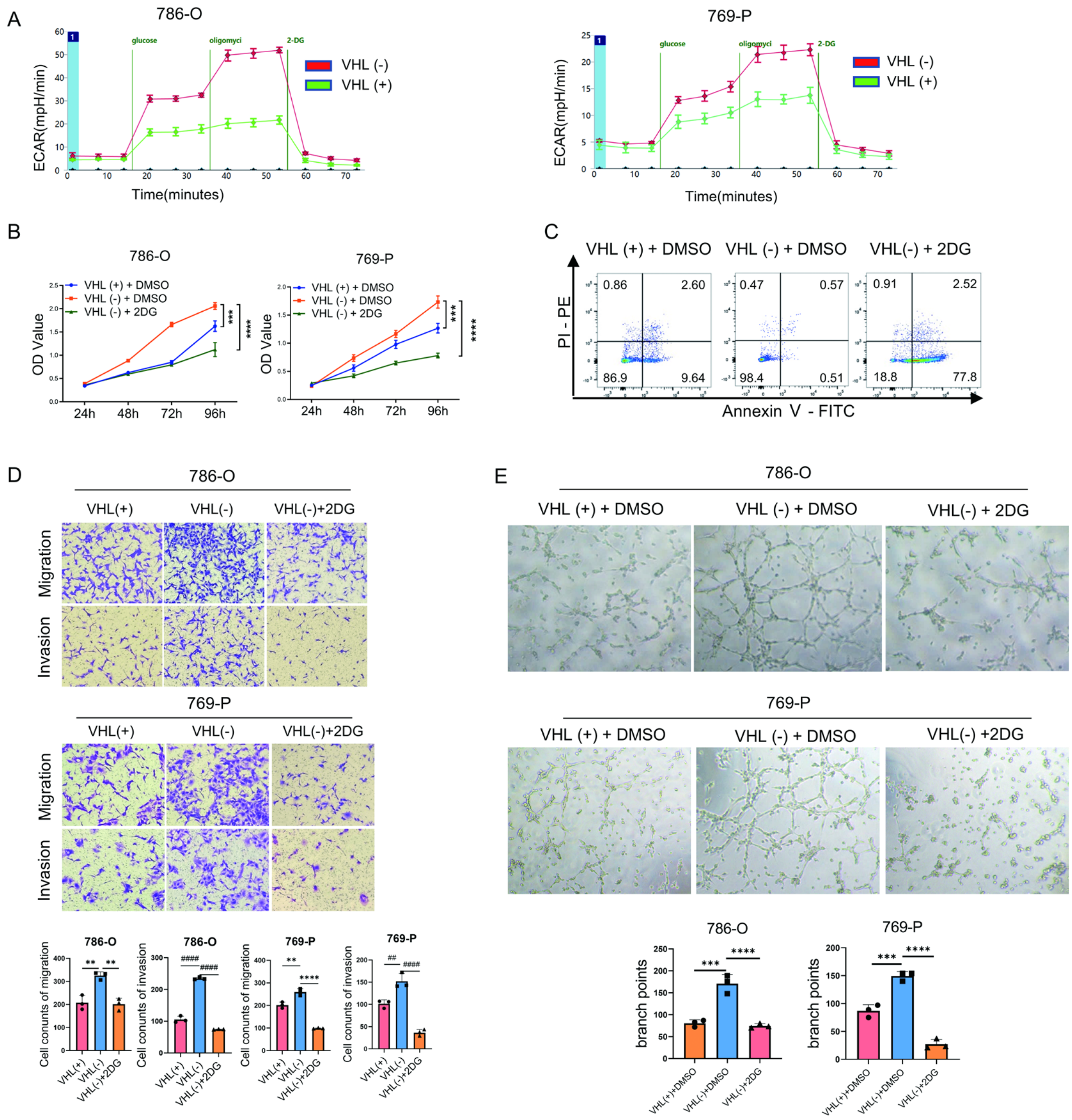

Figure S3. VHL loss–driven glycolysis fuels malignant behaviors in ccRCC. (A) ECAR analysis of glycolytic activity in VHL-deficient and VHL-reconstituted 786-O and 769-P cells. **(B)** CCK-8 analysis of cell proliferation in VHL-deficient and VHL-reconstituted 786-O and 769-P cells with or without 2-DG treatment. **(C)** Flow cytometry analysis of apoptosis using Annexin V/PI staining in VHL-deficient and

VHL-reconstituted 786-O cells with or without 2-DG treatment. **(D)** Transwell assays evaluating cell migration and invasion in VHL-deficient and VHL-reconstituted 786-O and 769-P cells with or without 2-DG treatment. **(E)** Tube formation assays performed with conditioned media from VHL-deficient and VHL-reconstituted 786-O and 769-P cells with or without 2-DG treatment. Data are presented as mean ± SD. Statistical analyses were performed using unpaired two-tailed Student's t-tests or one-way ANOVA, as appropriate. $^{**}p < 0.01$, $^{***}p < 0.001$, $^{****}p < 0.0001$. VHL (−): VHL-deficient; VHL (+): VHL-rescued.

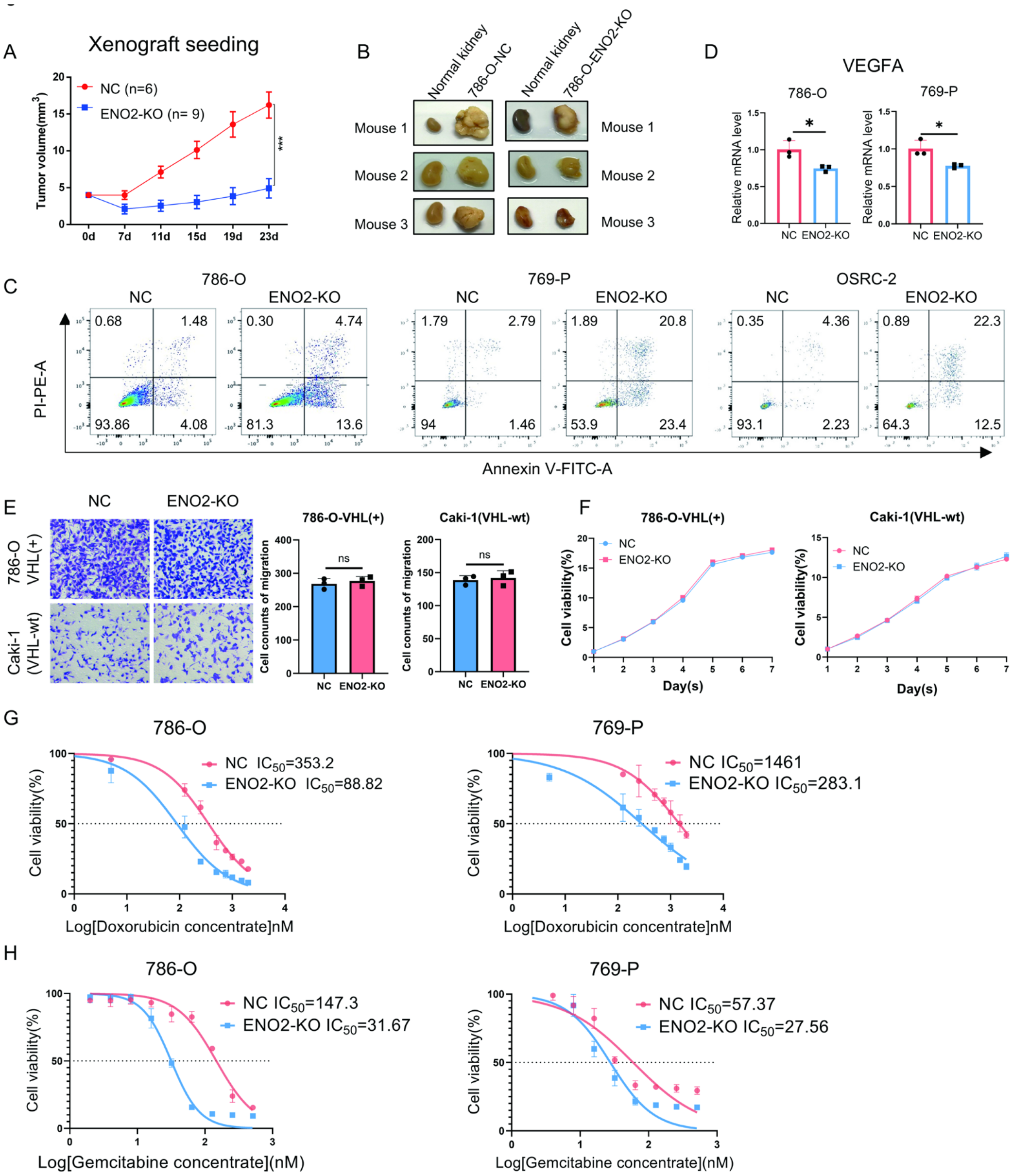

Figure S4. ENO2 ablation inhibits tumor growth and enhances chemo-sensitivity in ccRCC. (A) Xenograft seeding followed by tumor growth curve analysis to examine the effect of ENO2 ablation on the growth of seeded xenografts derived from a large 786-O tumor xenograft (n = 6 for NC, n = 9 for ENO2-KO). **(B)** Representative images of normal kidneys and ccRCC tissues from orthotopic xenograft mice bearing control or ENO2-KO 786-O cells. **(C)** Flow cytometry analysis of apoptosis in ENO2-ablated and control 786-O, 769-P, and OSRC-2 cells using Annexin V/PI staining. **(D)** RT-qPCR analysis of VEGFA mRNA expression in ENO2-ablated and control 786-O and 769-P cells. **(E)** Representative images and quantification of transwell migration assays in VHL-rescued 786-O cells and intrinsically VHL-wild-type Caki-1 cells following ENO2 knockout. **(F)** Cell growth curves of 786-O-VHL (+) and Caki-1 (VHL-wt) cells with or without ENO2 knockout. **(G)** IC_{50} determination to assess the effect of ENO2 ablation on chemo-sensitivity to doxorubicin in VHL-deficient ccRCC cells. **(H)** IC_{50} measurements to assess the effect of ENO2 ablation on chemo-sensitivity to Gemcitabine in VHL-deficient ccRCC cells. Data are presented as mean ± SD. Statistical analysis was performed using unpaired two-tailed Student's t-test. * $p < 0.05$, ** $p < 0.01$, *** $p < 0.001$, **** $p < 0.0001$; ns, not significant.

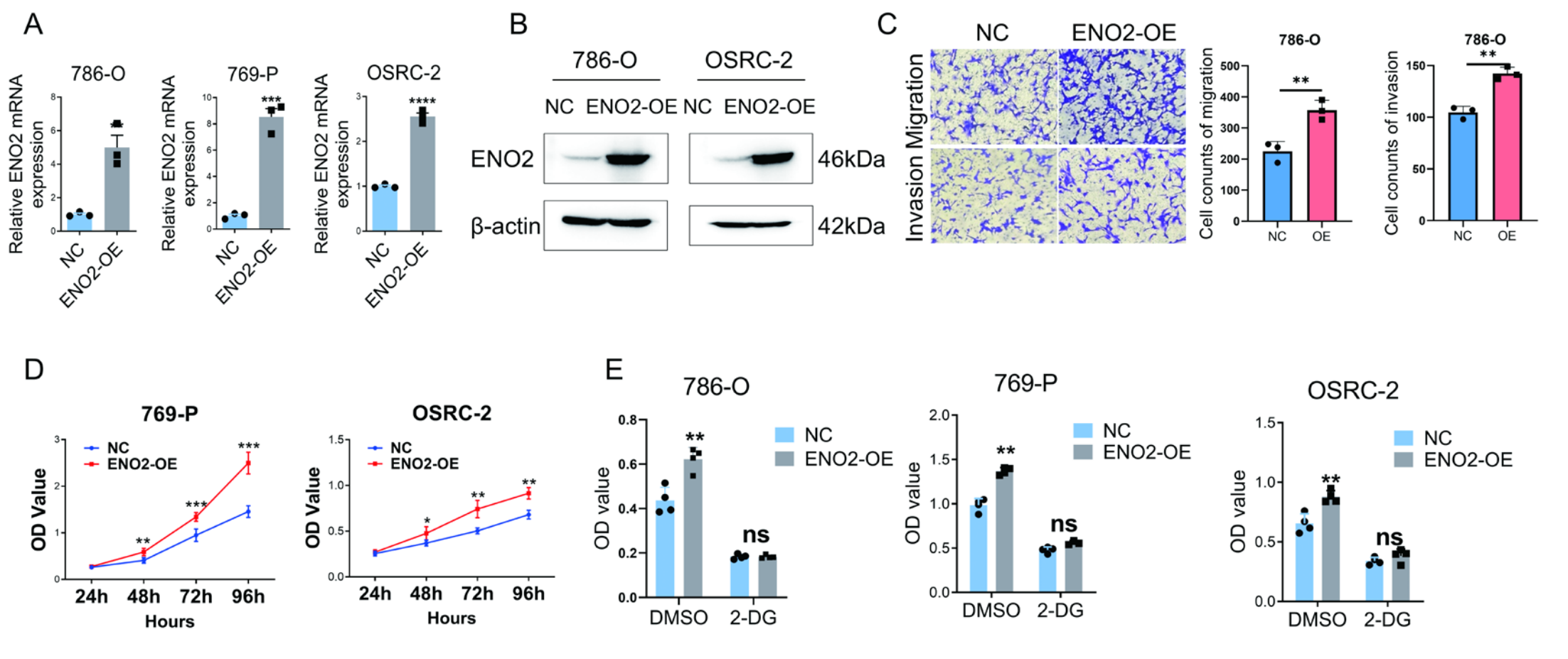

Figure S5. ENO2 overexpression promotes malignant phenotypes in ccRCC in a

glycolysis-dependent manner. (A) RT-qPCR analysis of ENO2 mRNA expression in ENO2-overexpressing (ENO2-OE) and control (NC) 786-O, 769-P, and OSRC-2 cells. **(B)** Western blot confirmation of ENO2 overexpression in 786-O and OSRC-2 cells. **(C)** Transwell assays evaluating cell migration and invasion in ENO2-OE and control 786-O cells. **(D)** CCK-8 analysis of cell proliferation in ENO2-OE and control ccRCC cells. **(E)** CCK-8 analysis of cell proliferation in ENO2-OE and control cells treated with vehicle (DMSO) or 2-DG for 48 hours in 786-O, 769-P, and OSRC-2 cells. Data are presented as mean ± SD. Statistical analysis was performed using unpaired two-tailed Student's t-test. *p < 0.05, **p < 0.01, ***p < 0.001, ****p < 0.0001; ns, not significant.

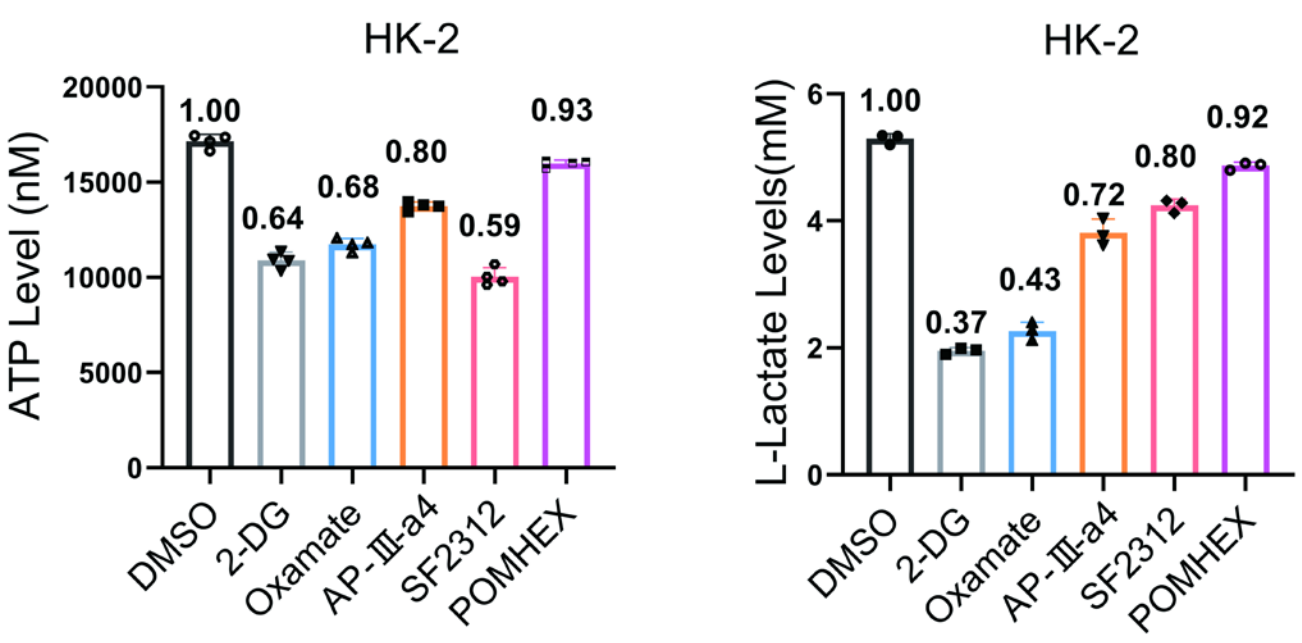

Figure S6. Effects of glycolysis inhibitors on ATP production and L-lactate levels in HK-2 cells. ATP and L-lactate measurements in HK-2 normal renal tubular epithelial cells treated with the indicated inhibitors. Numbers above the bars indicate values normalized to the DMSO control. Data are presented as mean ± SD.

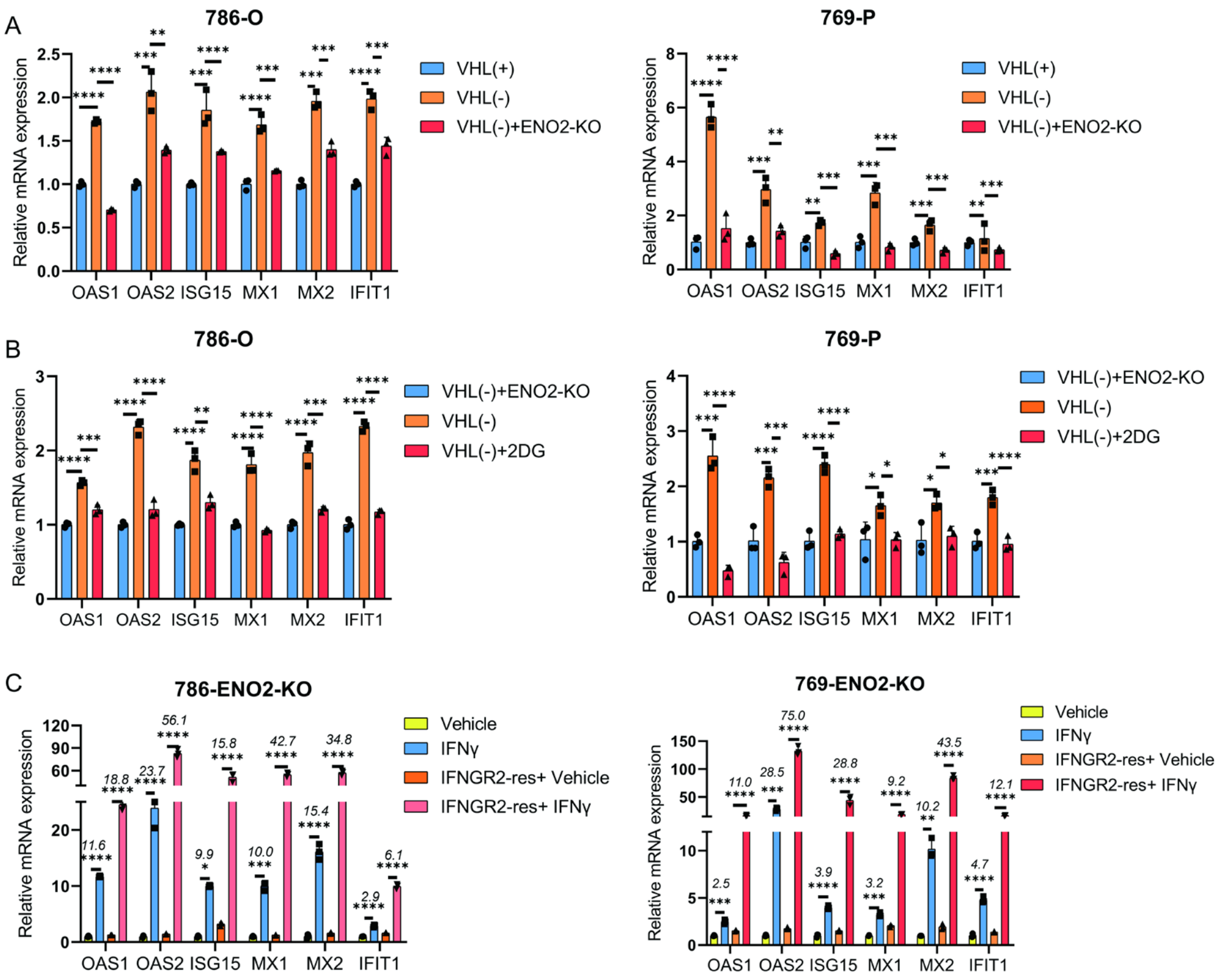

Figure S7. VHL loss promotes ISG expression through the HIF2α–ENO2–glycolysis–IFNGR2 axis in ccRCC cells. (A) RT-qPCR analysis of interferon-stimulated gene (ISG) expression in VHL-deficient, VHL-rescued, and VHL-deficient ENO2-knockout 786-O and 769-P cells. (B) RT-qPCR analysis of ISG expression in VHL-deficient 786-O and 769-P cells with ENO2 knockout or 2-DG treatment. (C) RT-qPCR analysis of ISG expression in ENO2-knockout 786-O and 769-P cells treated with vehicle or IFN-γ, with or without IFNGR2 restoration. Data are presented as mean ± SD. Statistical analysis was performed using one-way ANOVA followed by Tukey's multiple-comparisons test. **p* < 0.05, ***p* < 0.01, ****p* < 0.001, *****p* < 0.0001. VHL (−): VHL-deficient; VHL (+): VHL-rescued; IFNGR2-res: IFNGR2-restored.

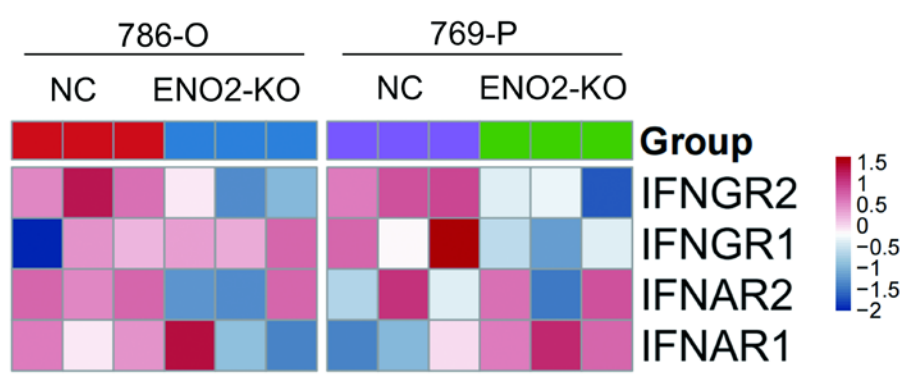

Figure S8. ENO2 regulates interferon signaling receptor expression in ccRCC cells. Heatmap analysis of RNA-seq data showing expression of interferon receptors (IFNGR2, IFNGR1, IFNAR2, and IFNAR1) in ENO2-KO and control (NC) 786-O and 769-P cells.

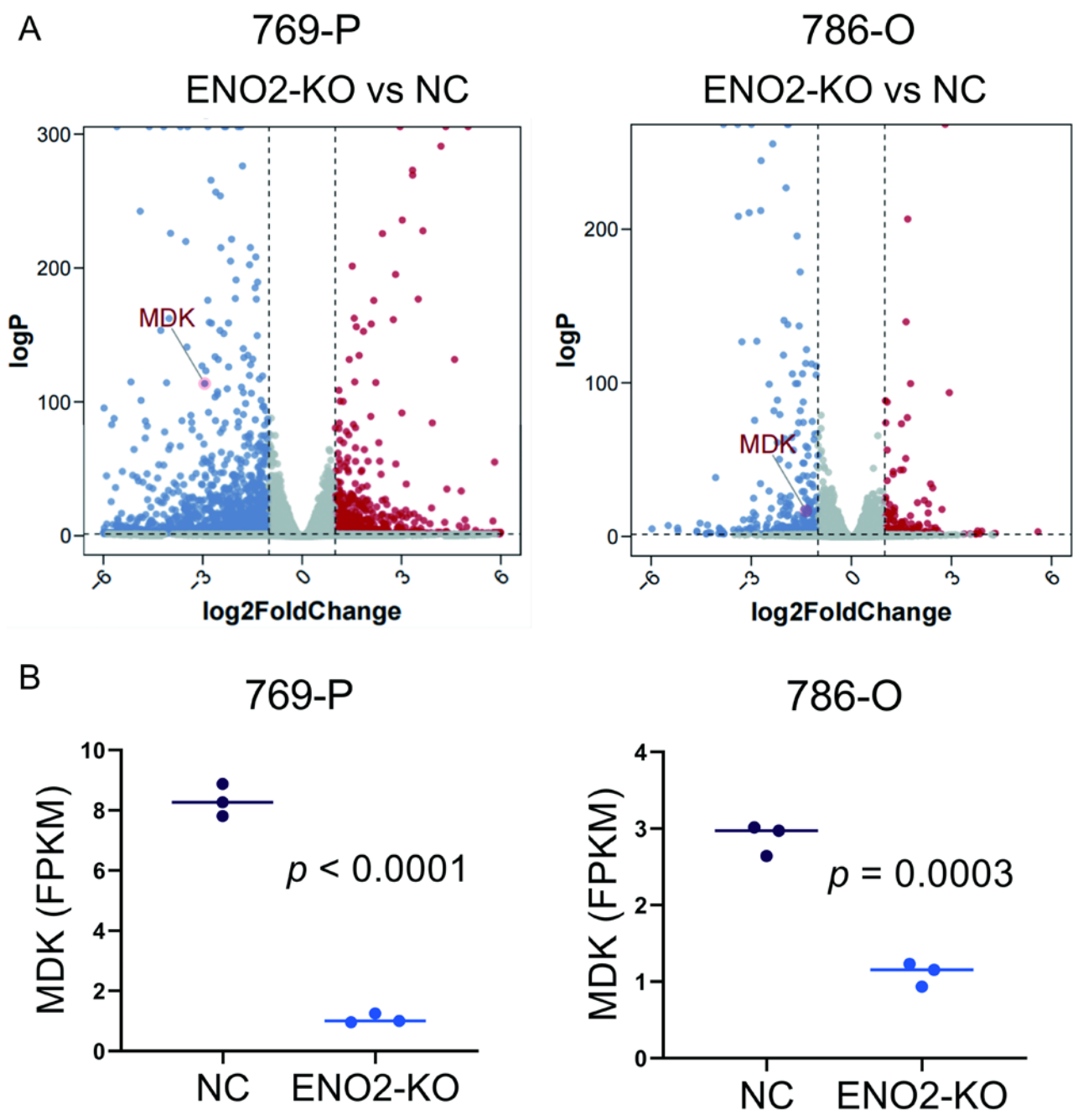

Figure S9. MDK is a downstream target of ENO2 in ccRCC cells. (A) Volcano plots showing the differentially expressed genes in ENO2-KO versus control in 769-P and 786-O cell lines, with MDK highlighted. **(B)** RNA-seq quantification of MDK expression (FPKM) in ENO2-KO and control 769-P and 786-O cells.

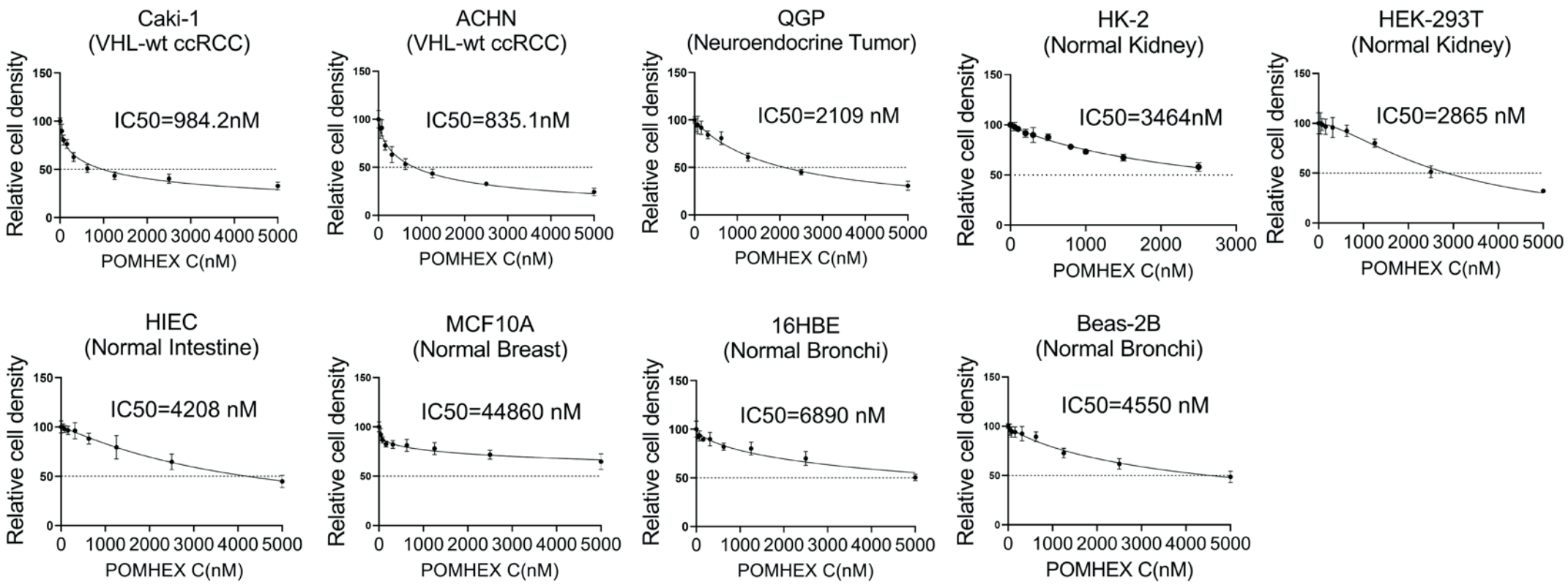

Figure S10. POMHEX sensitivity analysis. CCK-8 determination of the IC50 values of POMHEX in two VHL-wt ccRCC cell lines (Caki-1, ACHN), one neuroendocrine tumor cell line (QGP), and six normal cell lines (HK-2, HEK-293T, HIEC, MCF10A, 16HBE, Beas-2B). Data are presented as mean ± SD.

Table S1. Antibodies used in this study.

Antibody	brand	Cat No.	dilution ratio
ENO1	Proteintech	11204-1-AP	1:1000
ENO2	Proteintech	10149-1-AP	1:1000
MDK	Proteintech	11009-1-AP	1:1000
VHL	CST	68547	1:1000
E-Cadherin	CST	3195	1:1000
N-Cadherin	CST	13116	1:1000
Snail	CST	3879	1:1000
Slug	CST	9585	1:1000
IFNGR2	Proteintech	10266-1-AP	1:1000
VEGFA	Proteintech	66828-1-Ig	1:1000
Pan-Kla	Jingjie PTM BioLab	PTM-1041RM	1:1000
H3K18la	Jingjie PTM BioLab	PTM-1406RM	1:1000
β-tubulin	Proteintech	10094-1-AP	1:2000
β-actin	Proteintech	66009-1-Ig	1:2000

Table S2. Primers used in this study.

Gene	Sequence (5'-3')
ENO1 forward	TGGTGTCTATCGAAGATCCCTT
ENO1 reverse	CCTTGGCGATCCTCTTTGG
ENO2 forward	AGGTGCAGAGGTCTACCATAC
ENO2 reverse	AGCTCCAAGGCTTCACTGTTC
ENO3 forward	TATCGCAATGGGAAGTACGATCT

ENO3 reverse	AAGCTCTTATACAGCTCTCCGA
MDK forward	CGCGGTCGCCAAAAAGAAAG
MDK reverse	TACTTGCAGTCGGCTCCAAAC
VEGFA forward	AACCATGAACTTTCTGCTGTCTTGG
VEGFA reverse	ATCAGGGTACTCCTGGAAGATGTCC
IFNGR2 forward	GCAGGCTTCCCAATGGATTTC
IFNGR2 reverse	CCCGACAGTCACATTCCGATA
IFNAR2 forward	ACCACTCCATTGTACCAACTCA
IFNAR2 reverse	TGTGCTTCTCCACTCATCTGT
E-Cadherin forward	CGAGAGCTACACGTTCACGG
E-Cadherin reverse	GGGTGTCGAGGGAAAAATAGG
N-Cadherin forward	AGCCAACCTTAACTGAGGAGT
N-Cadherin reverse	GGCAAGTTGATTGGAGGGATG
Snail forward	TCGGAAGCCTAACTACAGCGA
Snail reverse	AGATGAGCATTGGCAGCGAG
Slug forward	CGAACTGGACACACATACAGTG
Slug reverse	CTGAGGATCTCTGGTTGTGGT
OAS1 forward	AACTACCAGCAACTCTGCATCT
OAS1 reverse	GTCTCCACCACCCAAGTTTC
OAS2 forward	GCTCAGAAGCTGGGTTGGTT
OAS2 reverse	TTTCCGTCCATAGGAGCCAC
MX1 forward	AGCAAATCAAGGCACTGGAAGAG
MX1 reverse	TTGGCGGTTCTGTGGAGGTTA
MX2 forward	TGGCAGAGGCAACCAAGAAA
MX2 reverse	CCTTCTAACAACGGGAGCGA

ISG15 forward	AGCGAACTCATCTTTGCCAGTA
ISG15 reverse	CAGCATCTTCACCGTCAGGTC
IFIT1 forward	TGGGTATGCGATCTCTGCCT
IFIT1 reverse	TCAGGGCAAGGAGAACCTTAAT
β-actin forward	CATGTACGTTGCTATCCAGGC
β-actin reverse	CTCCTTAATGTCACGCACGAT
