# Supplementary material for: Targeting enolase isoenzyme 2 enables precision glycolysis inhibition in VHL-mutant renal cell carcinoma
Source: Cell Death Dis. 2026 Jul 14;17(1):872. doi: 10.1038/s41419-026-09102-4 (PMC13645809; doi:10.1038/s41419-026-09102-4)
Supplement: Supplementary file 2 [file 41419_2026_9102_MOESM2_ESM.pdf]

Fig.1 D

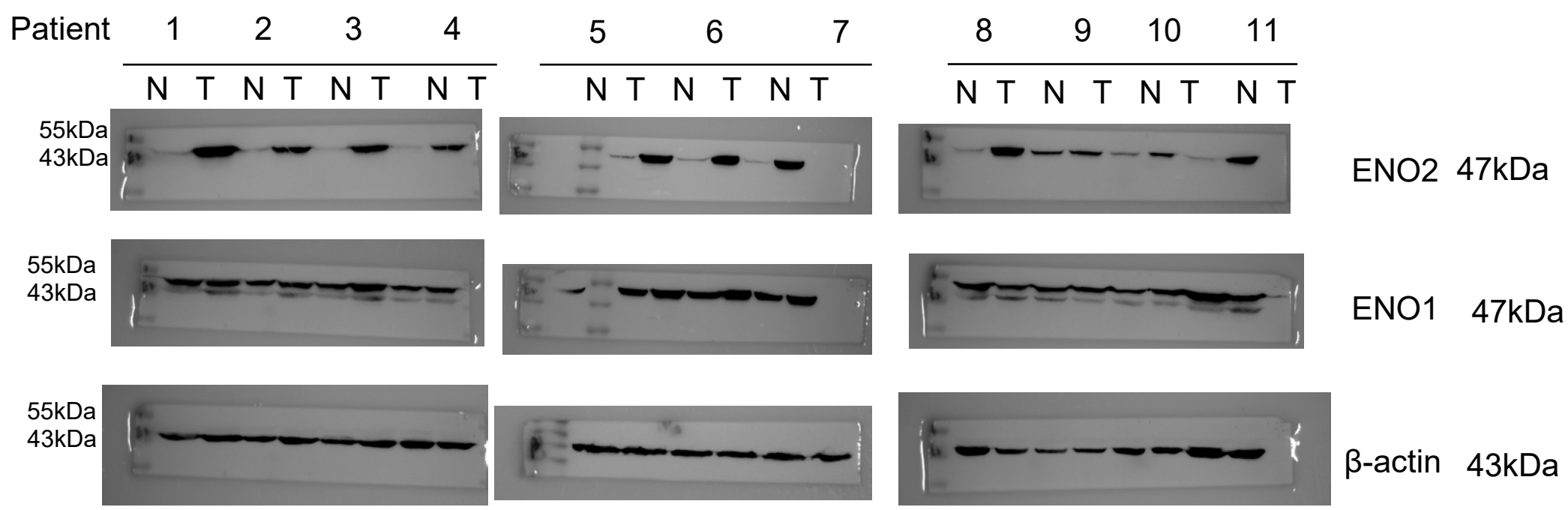

Patient
1
2
3
4
5
6
7
8
9
10
11
N T N T N T N T
N T N T N T
N T N T N T N T
55kDa
43kDa
ENO2 47kDa
ENO1 47kDa
β-actin 43kDa

Fig.2 C

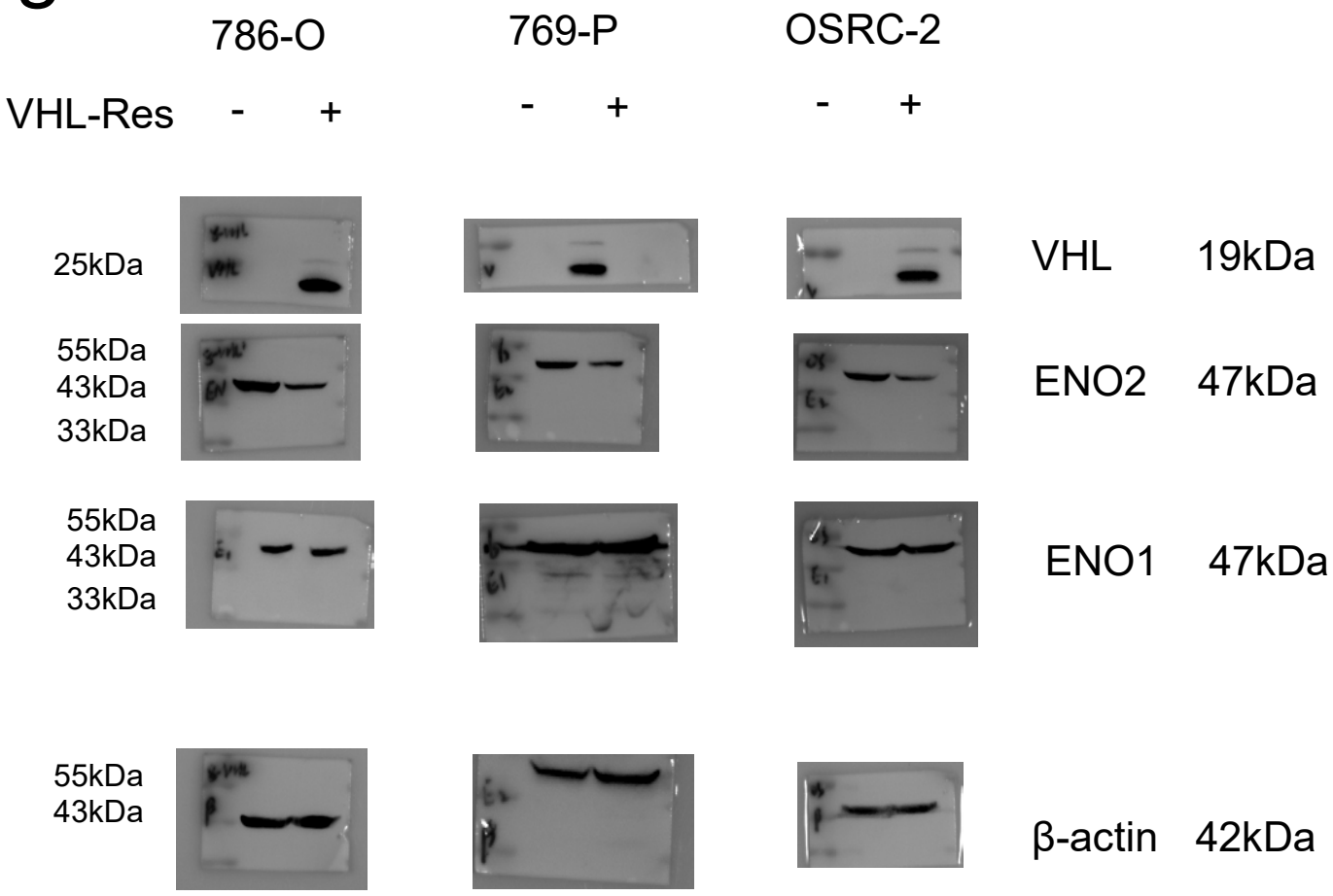

Fig.2 F

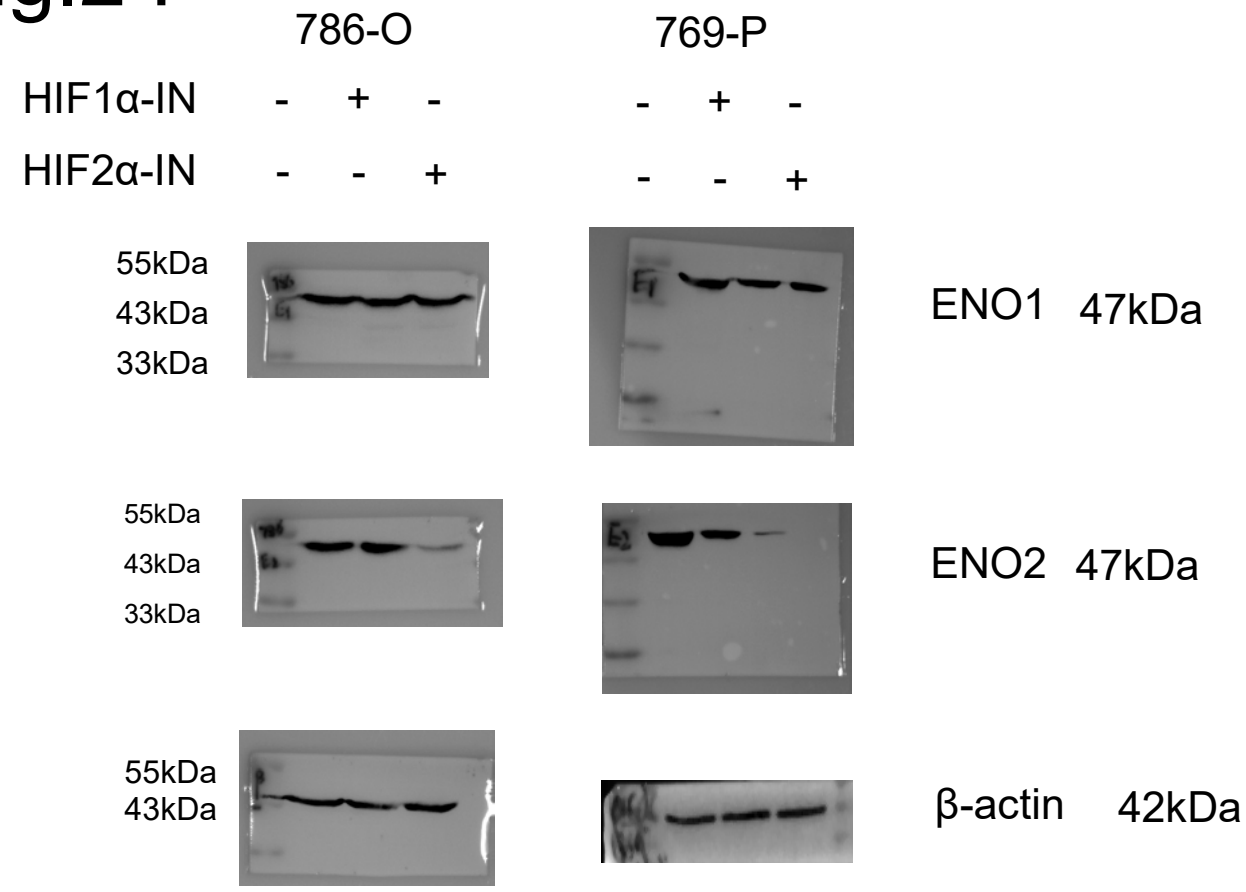

Fig.2 G

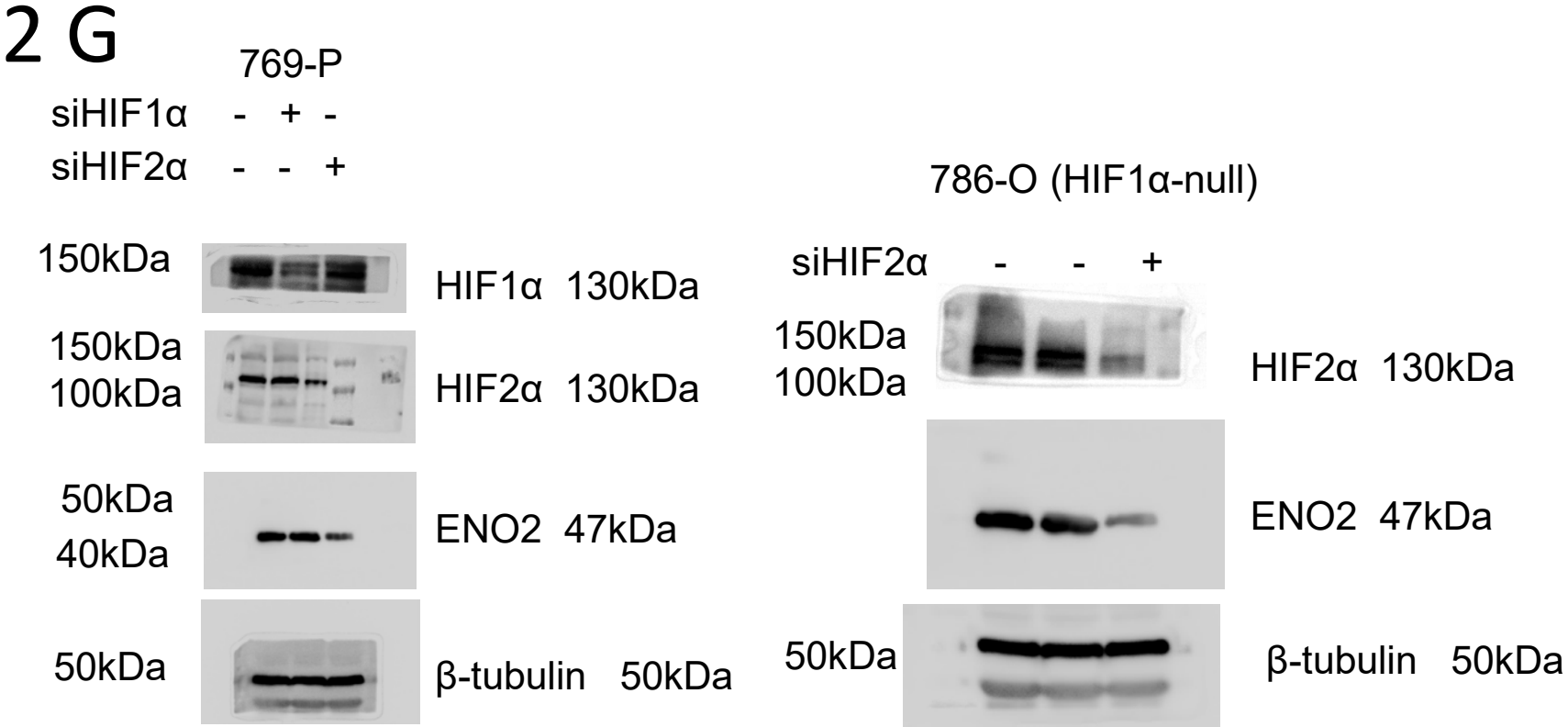

Fig.2 H

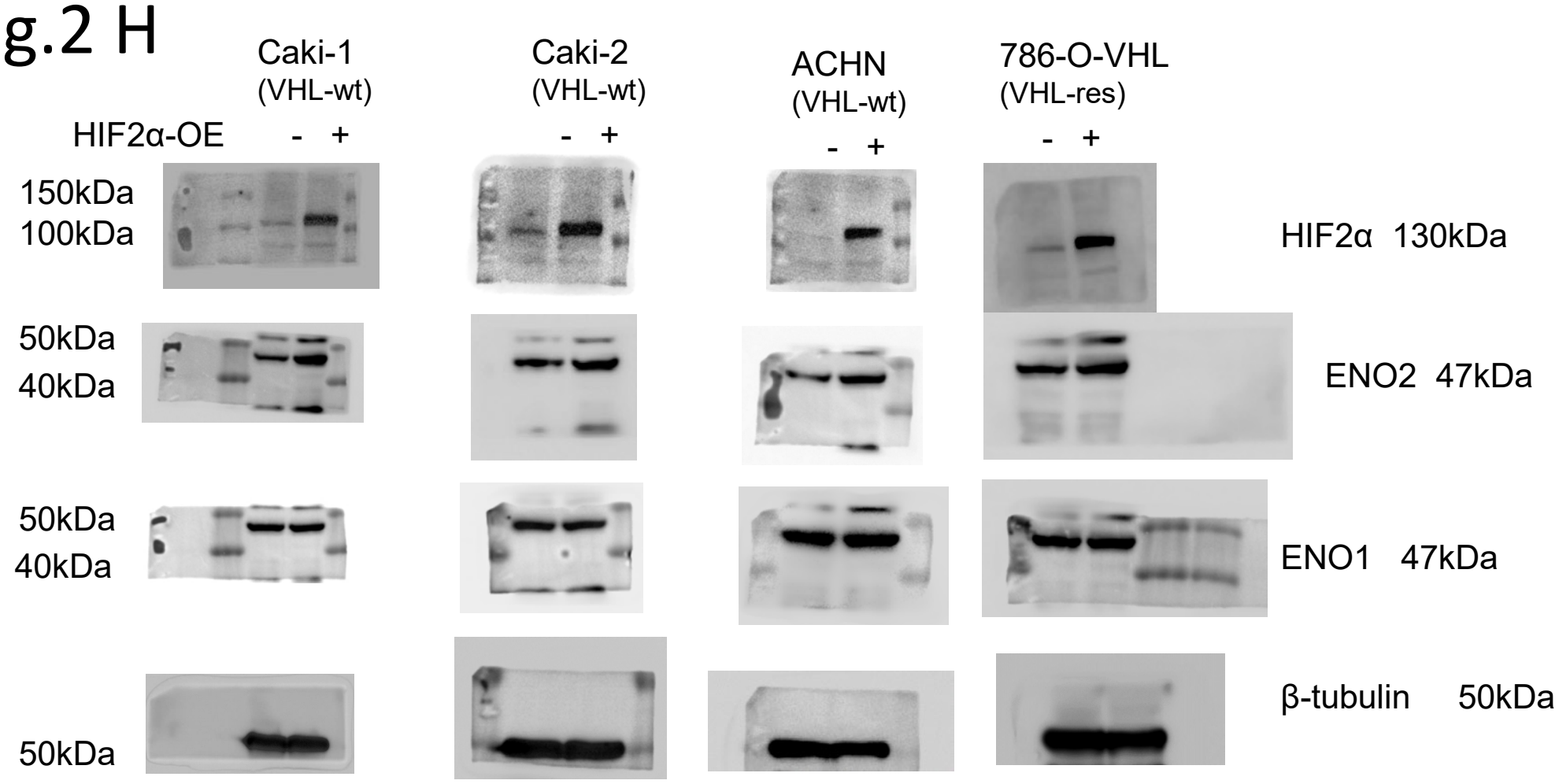

Fig.3J

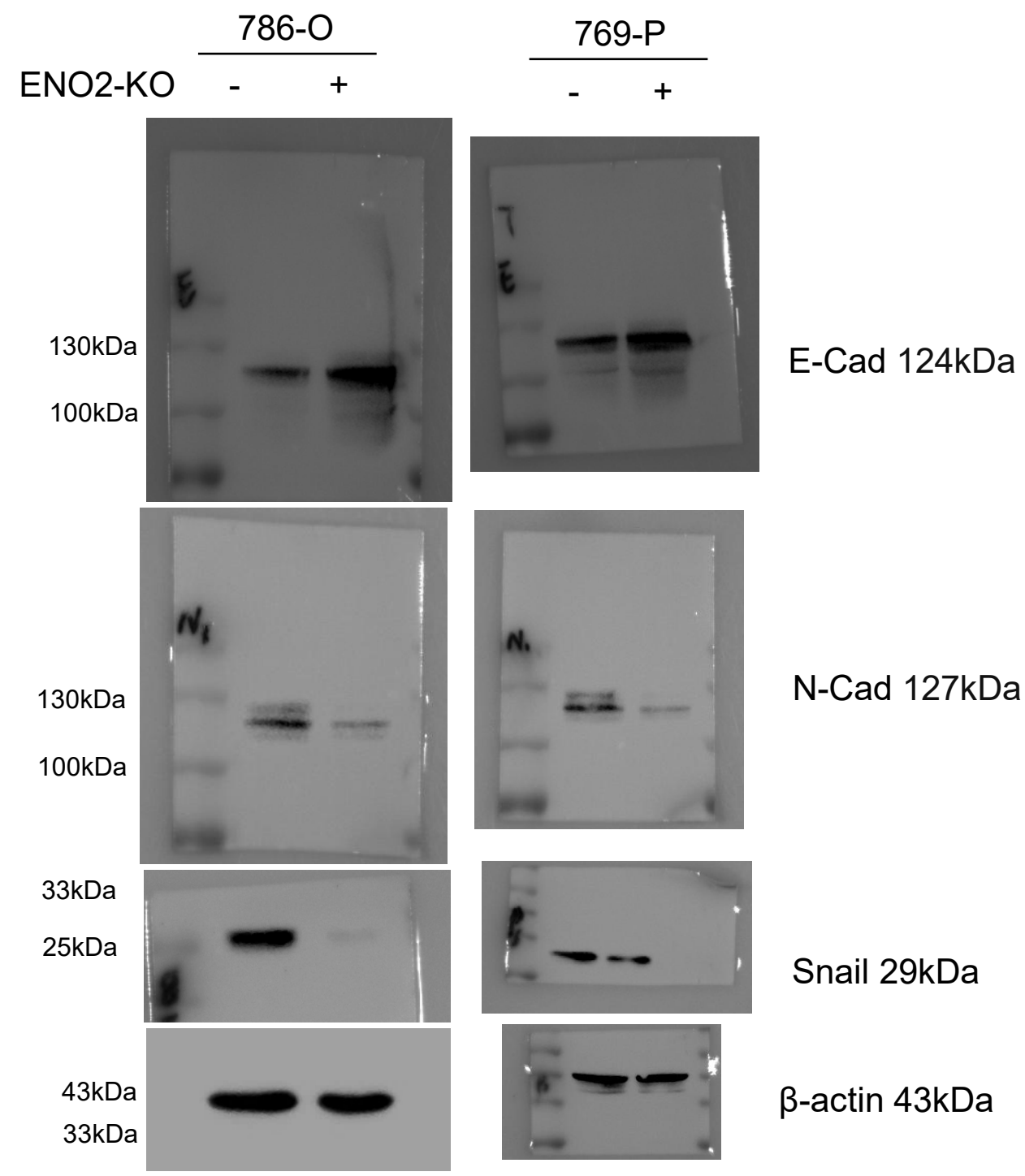

Fig.3A

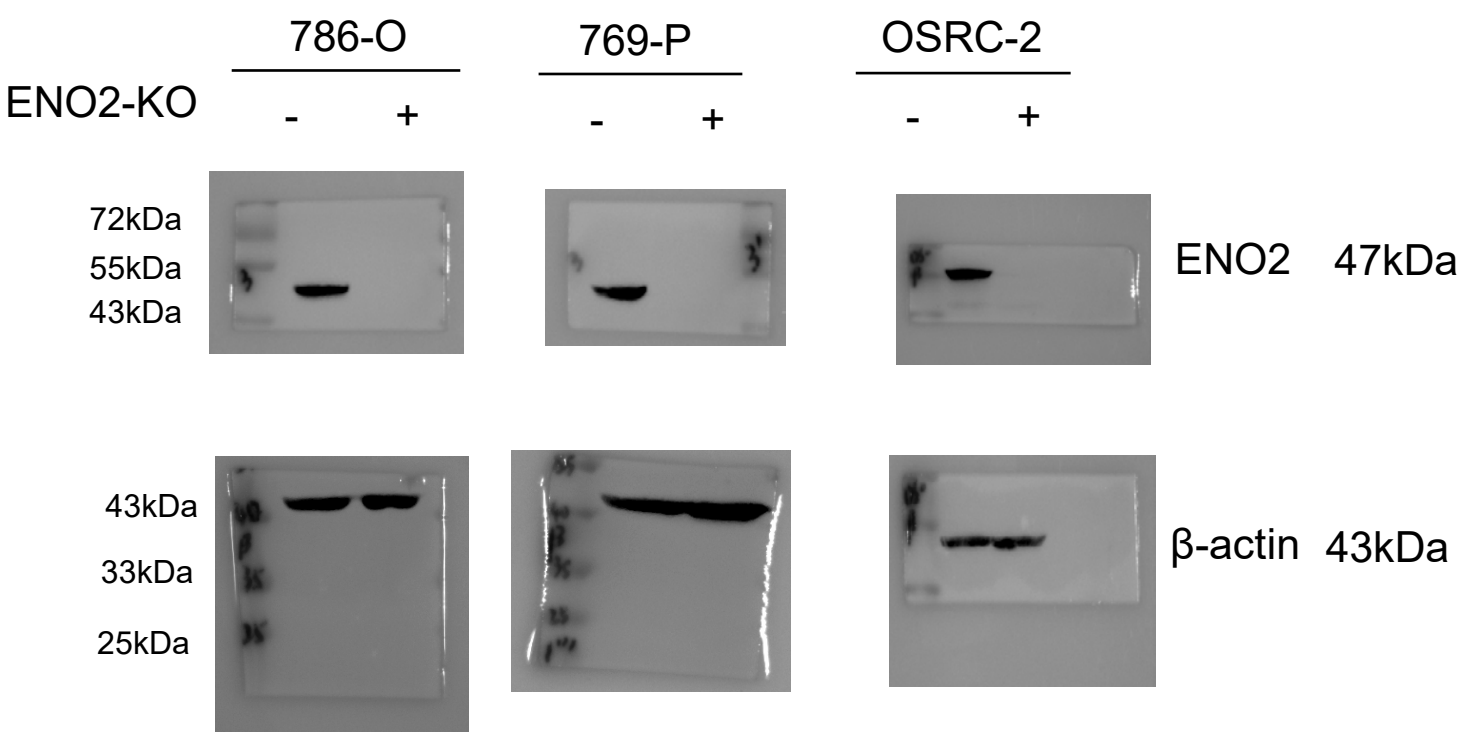

Fig.3M

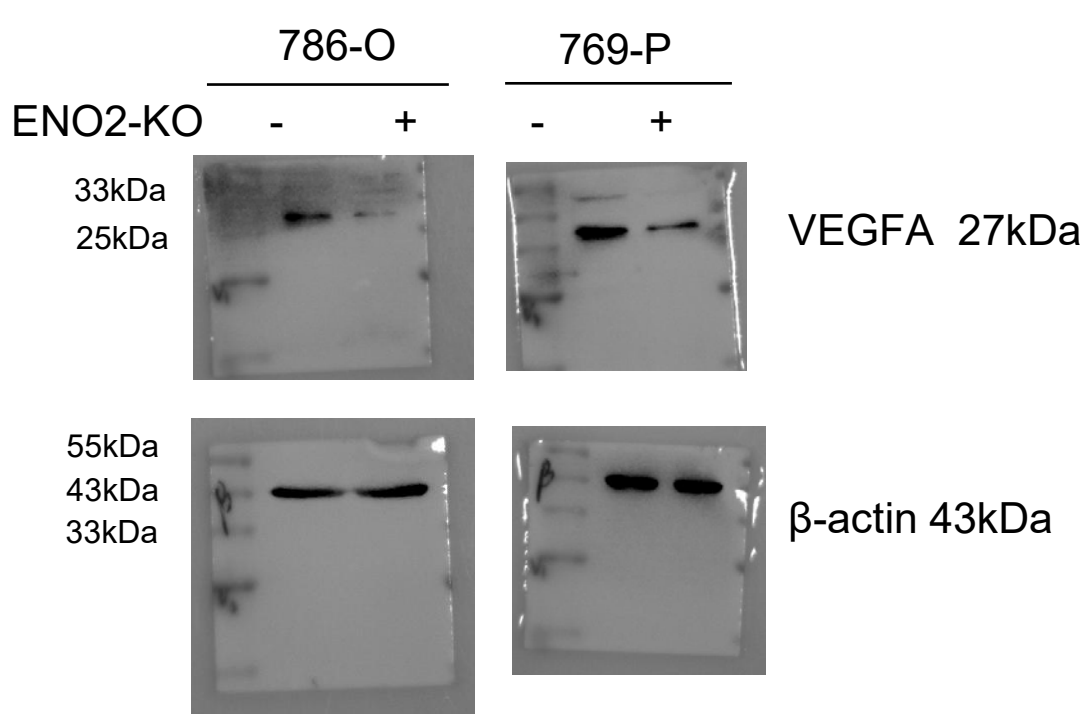

Fig.5E

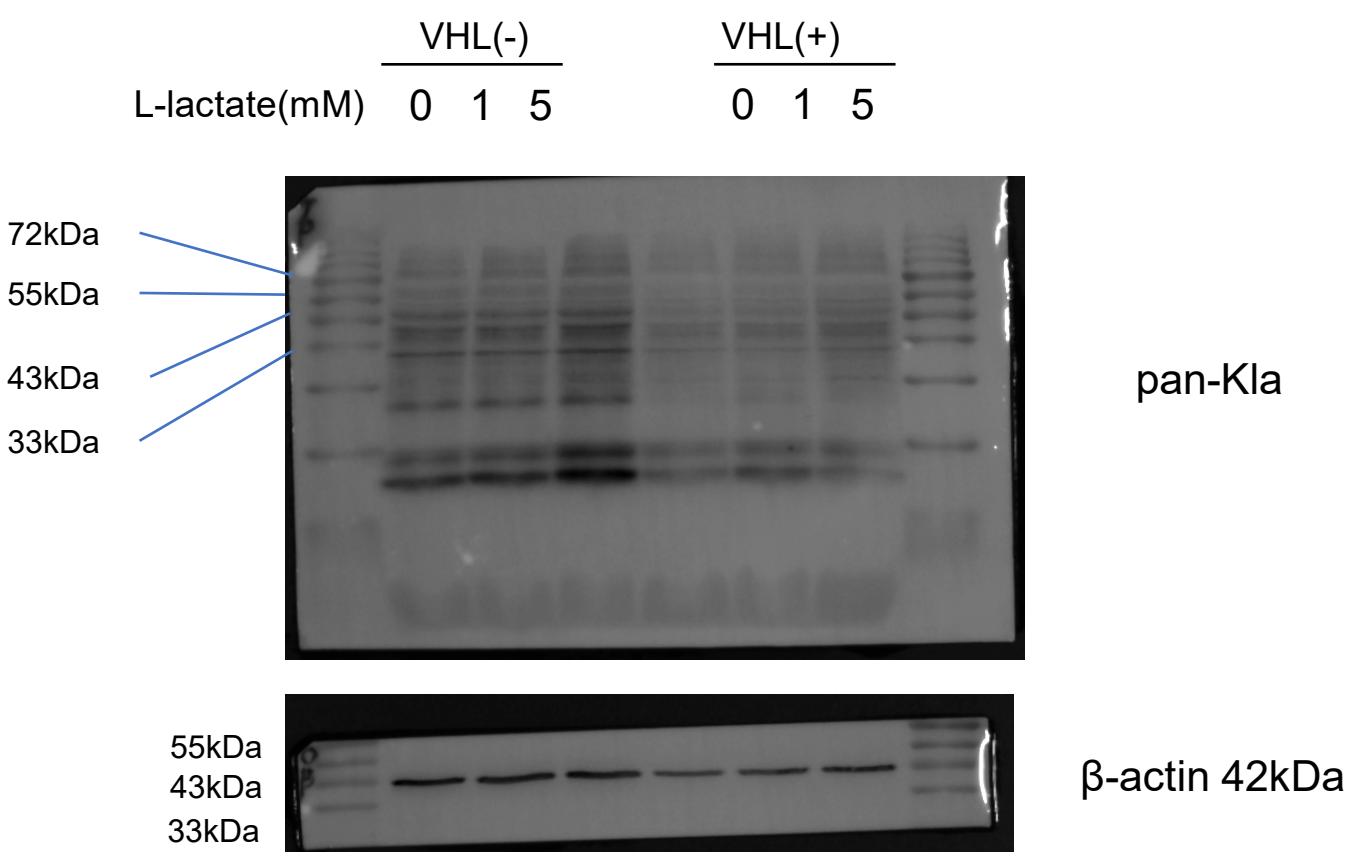
VHL(-)
VHL(+)
L-lactate(mM) 0 1 5 0 1 5
72kDa
55kDa
43kDa
33kDa
pan-Kla
55kDa
43kDa
33kDa
β-actin 42kDa

Fig.5F

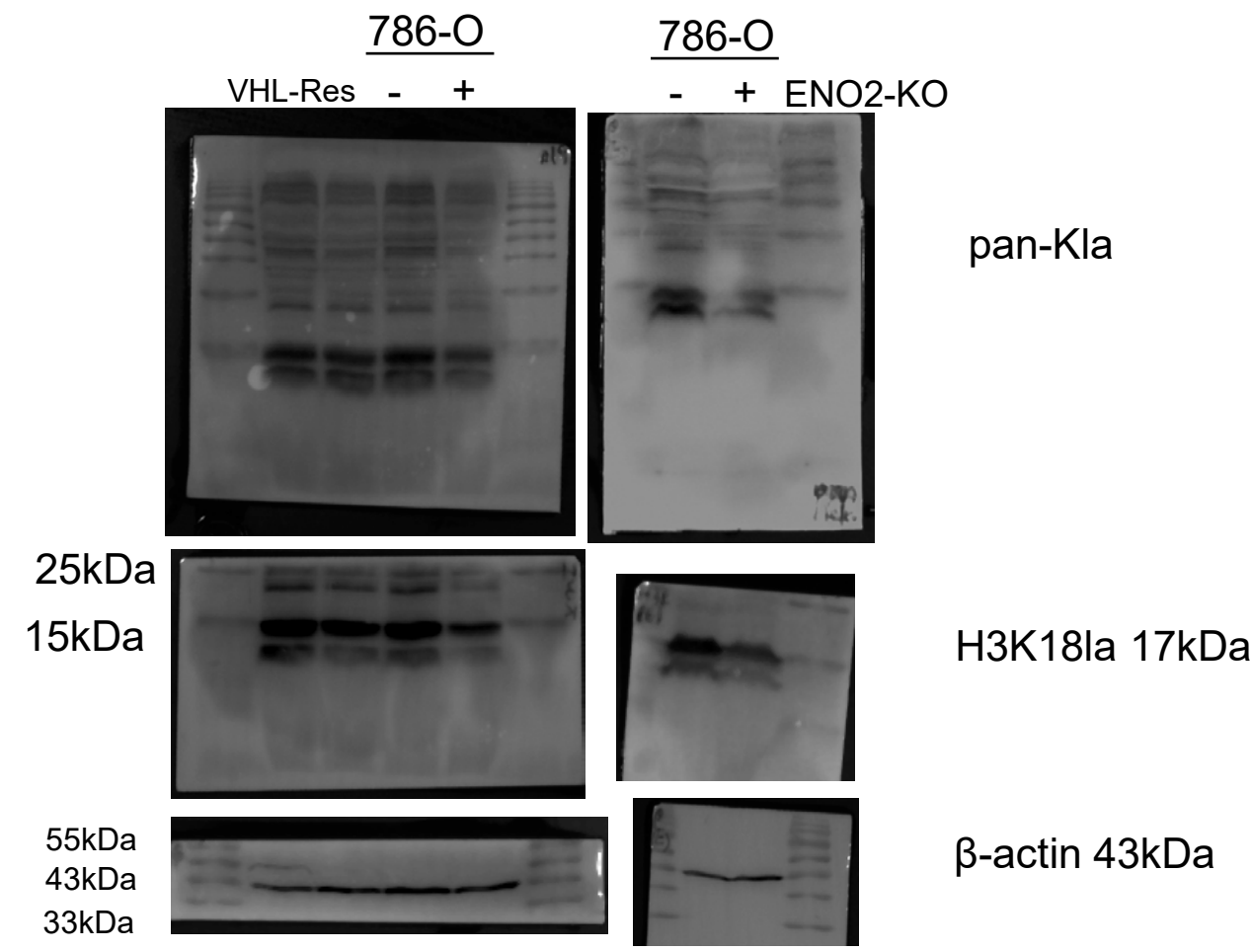
786-O
786-O
VHL-Res - +
- + ENO2-KO
pan-Kla
25kDa
15kDa
H3K18la 17kDa
55kDa
43kDa
33kDa
β-actin 43kDa

Fig.6A

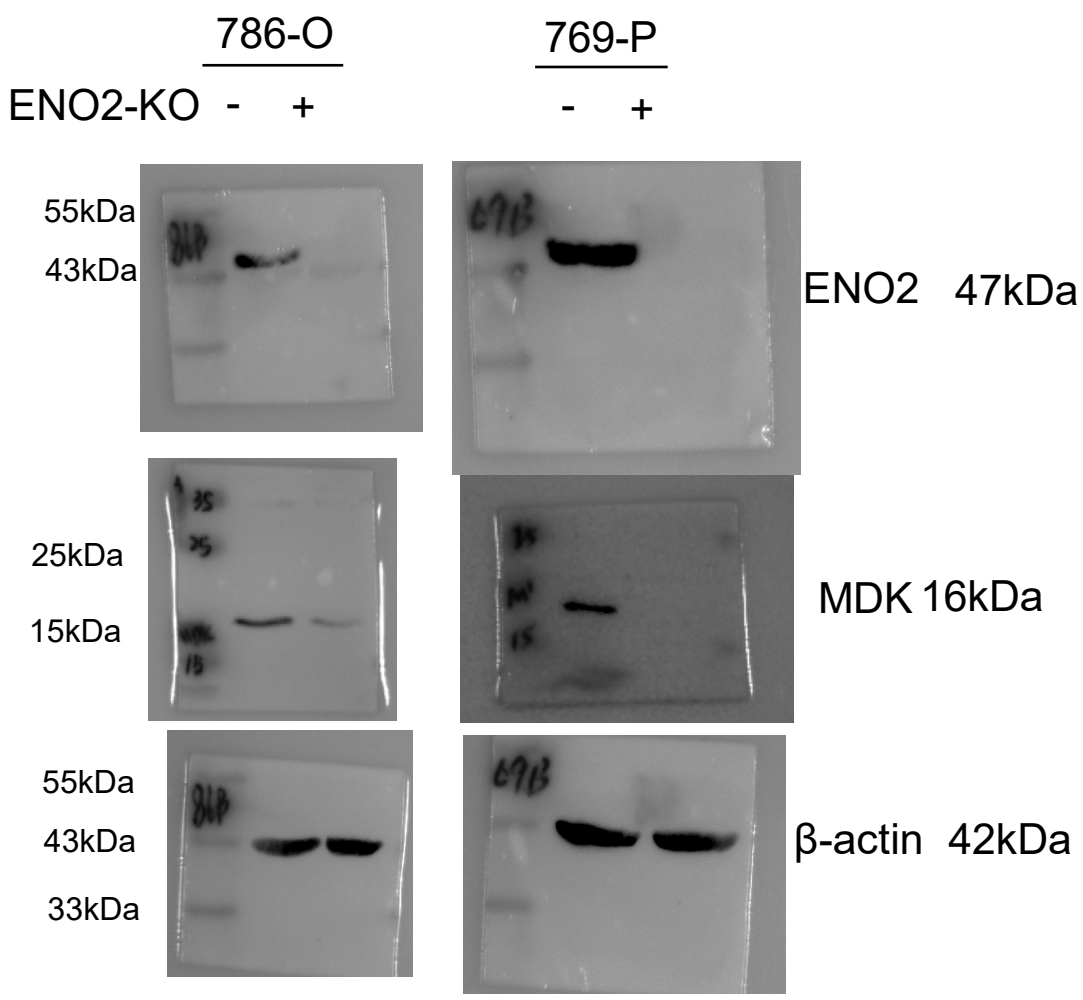

Fig.6B

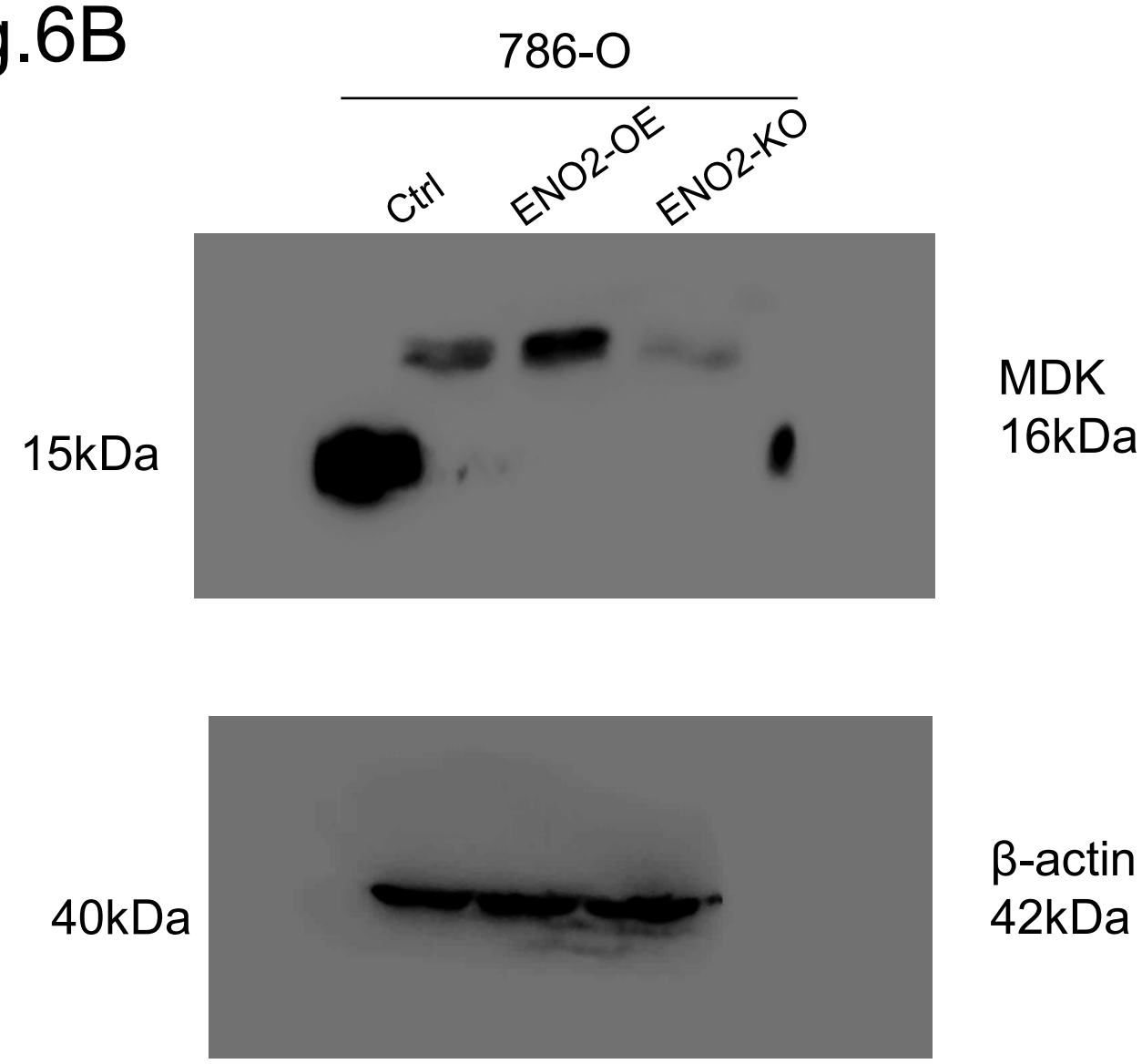

Fig.6C

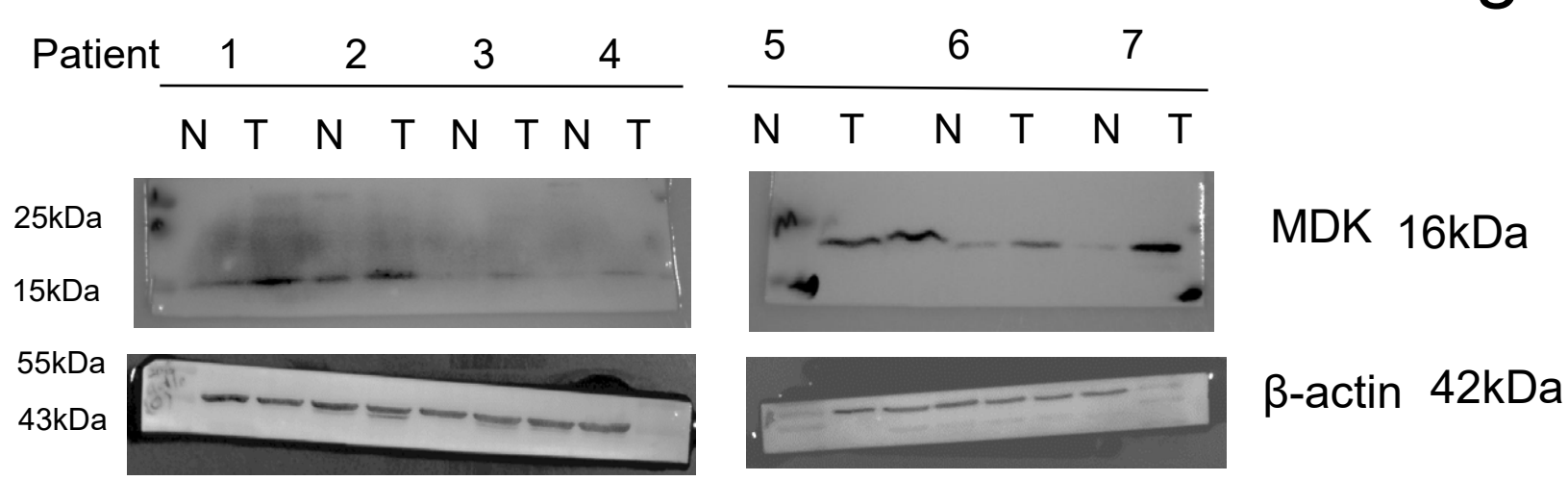

Fig.6G

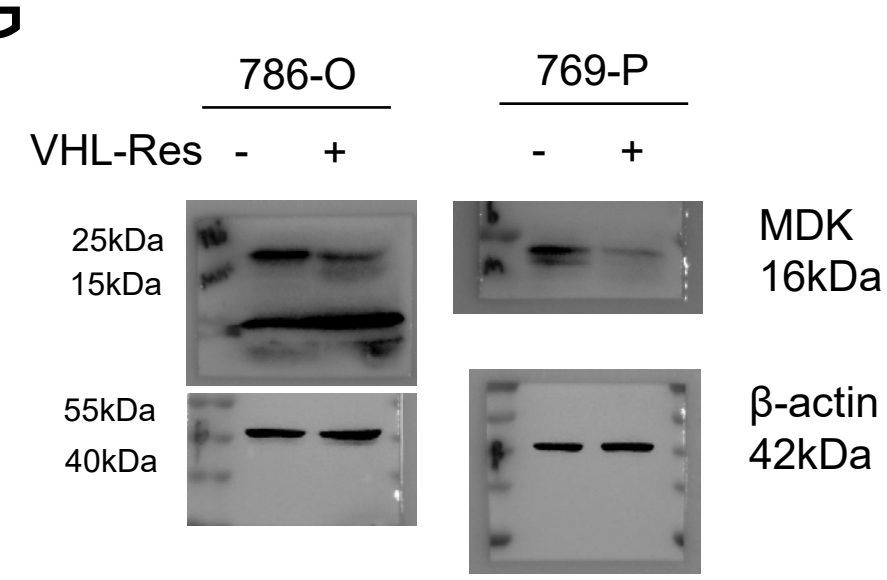

Fig.6I

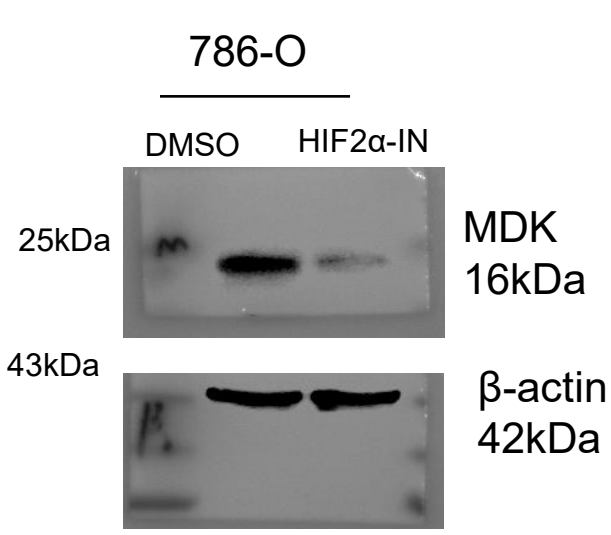

Fig.6J

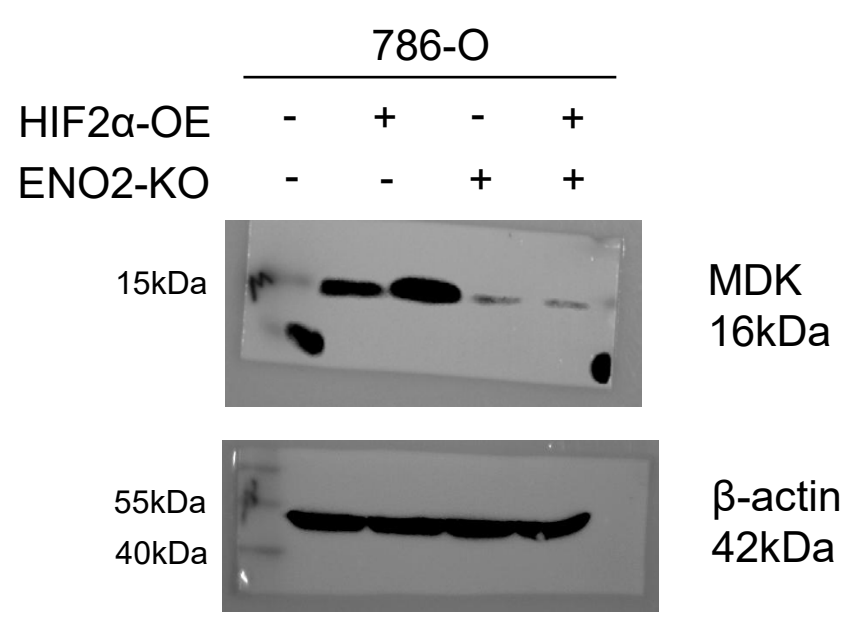

Fig.7A

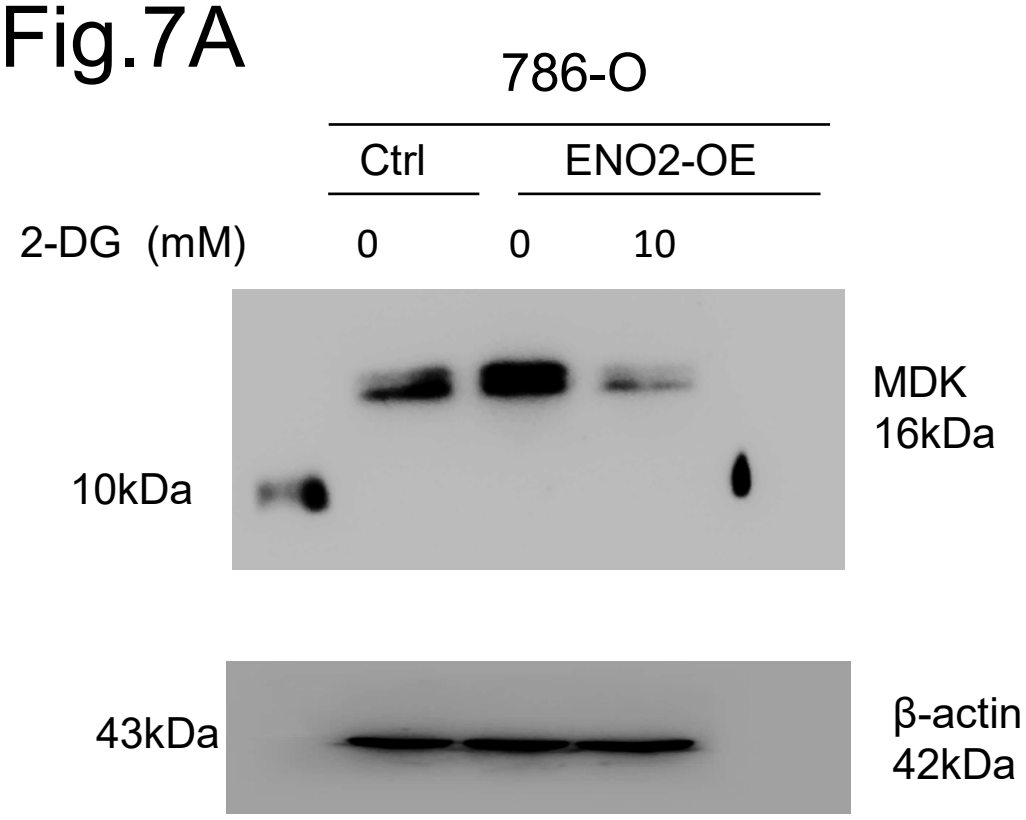

Fig.7B

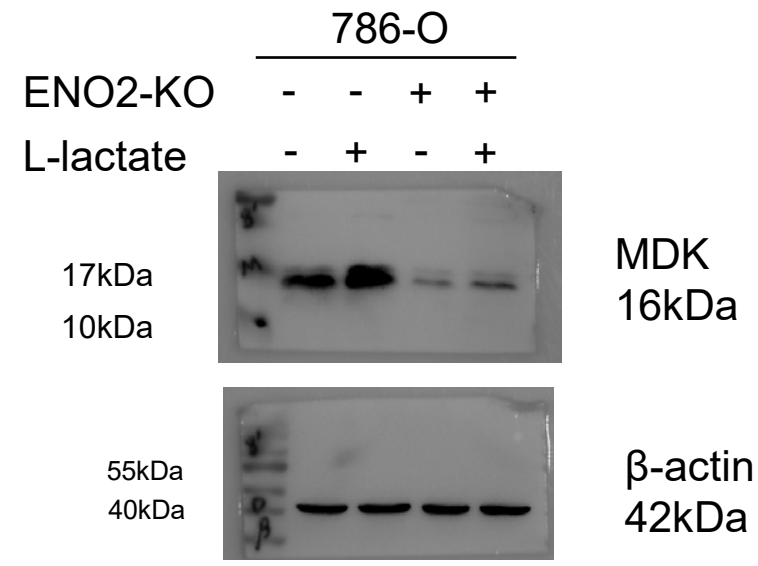

Fig.7C

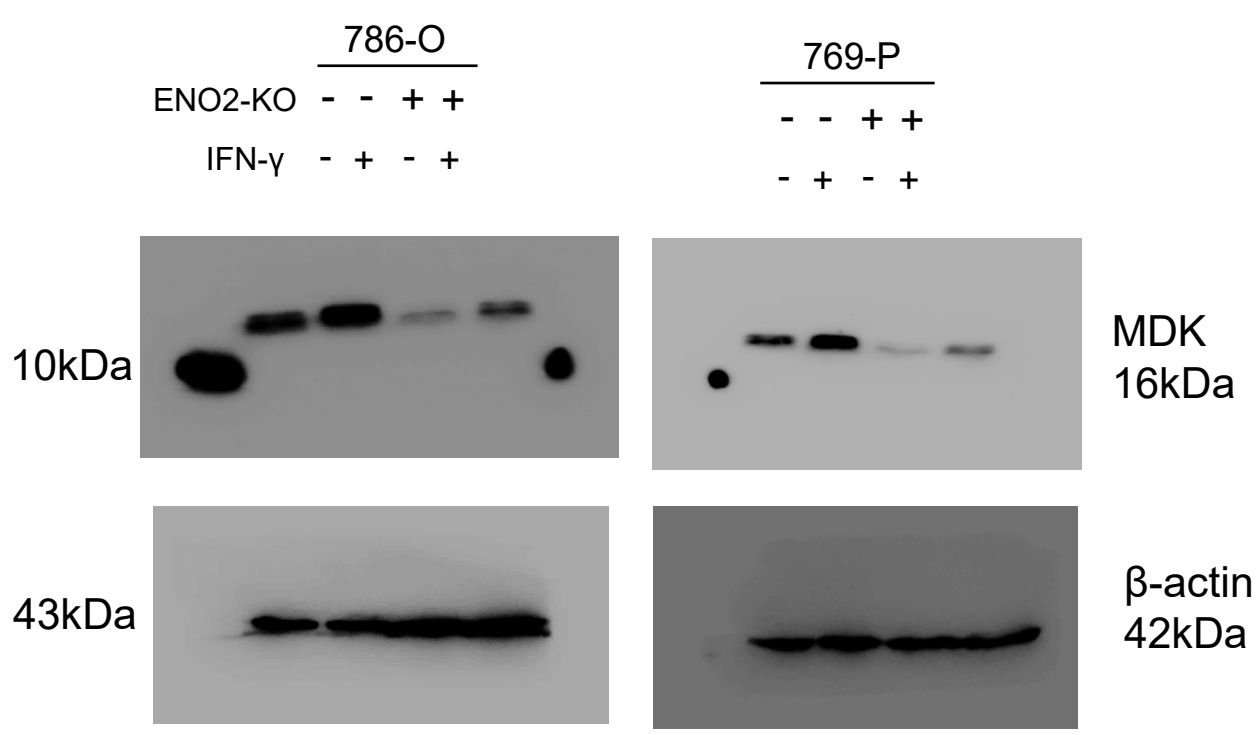

Fig.7D

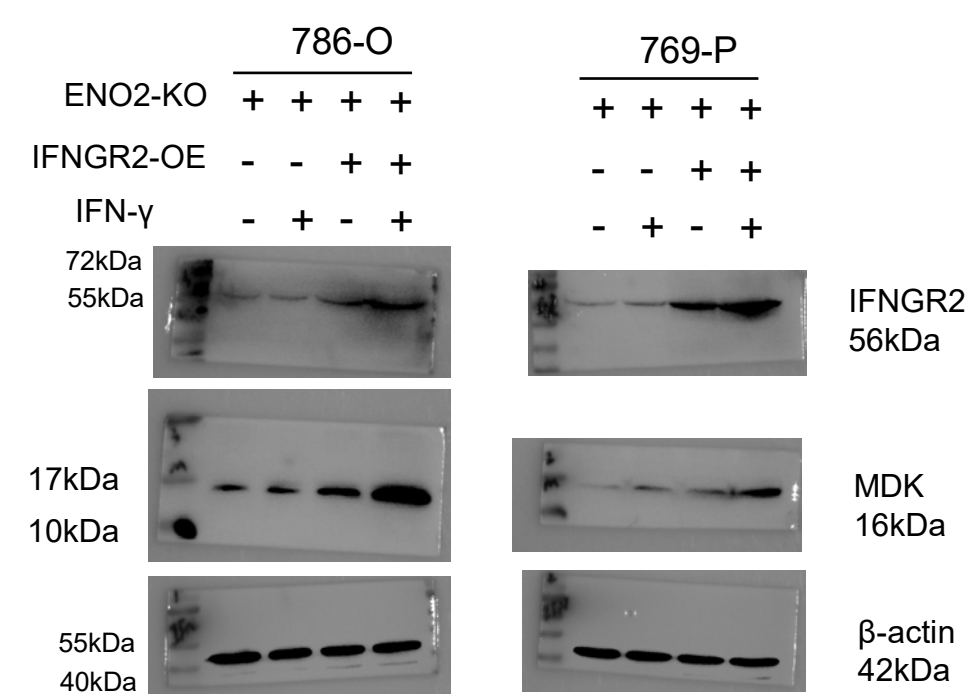

Fig.7H

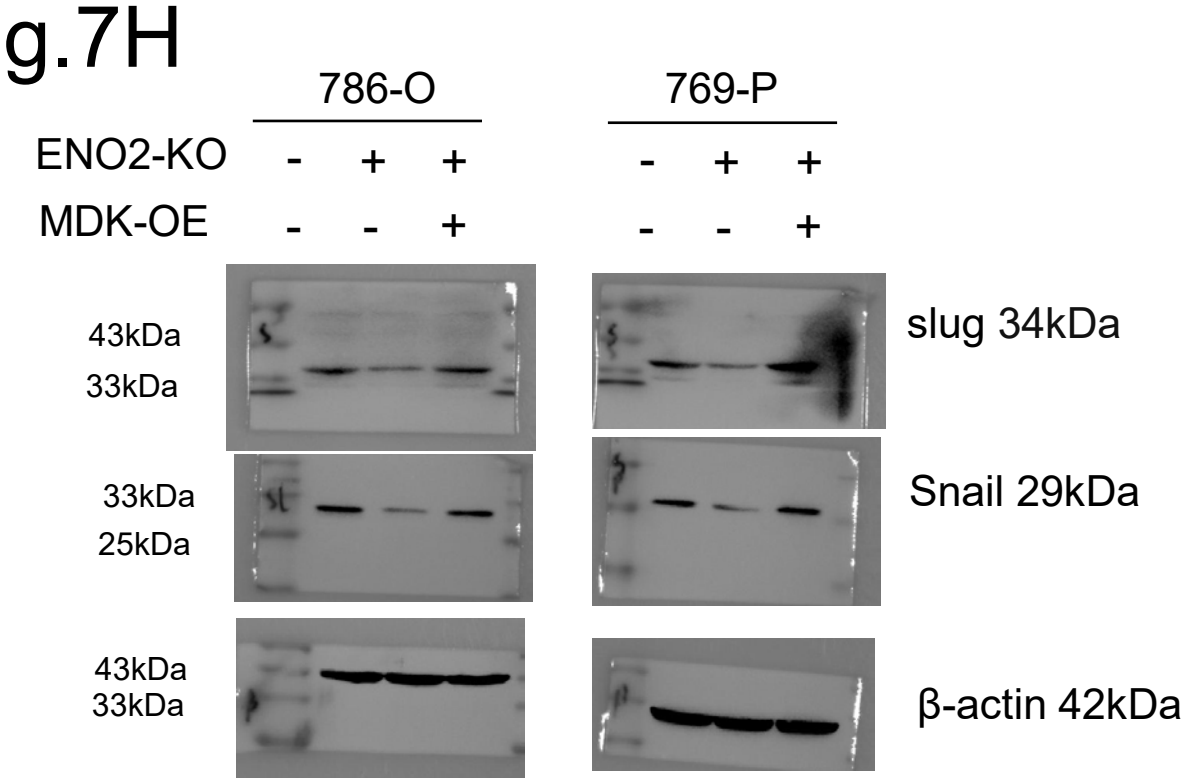

Fig.8B

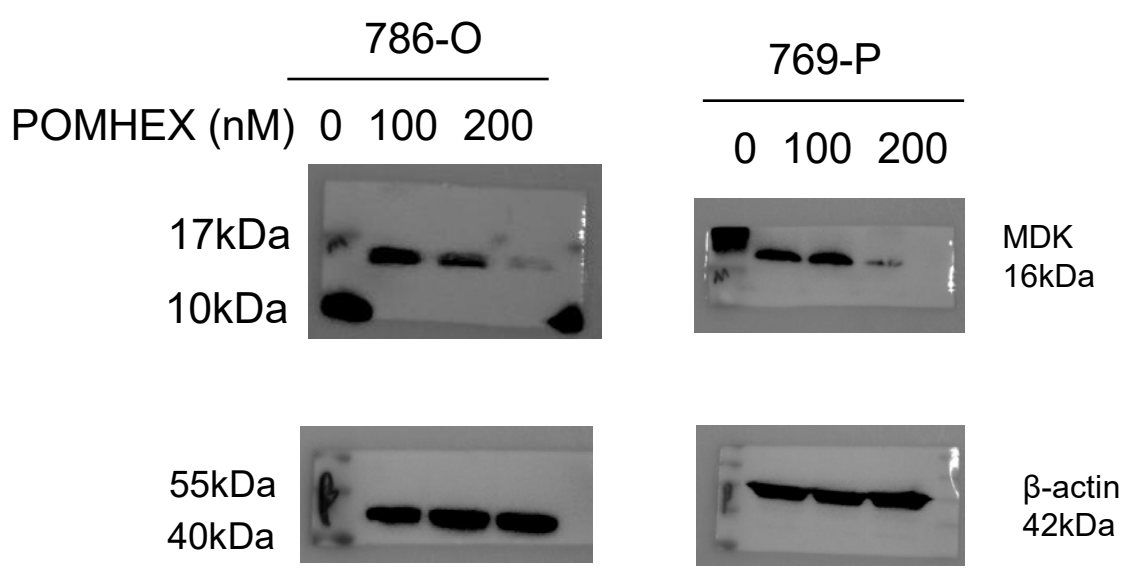
786-O
769-P
POMHEX (nM) 0 100 200
0 100 200
17kDa
10kDa
MDK
16kDa
55kDa
40kDa
β-actin
42kDa

Fig.S5B

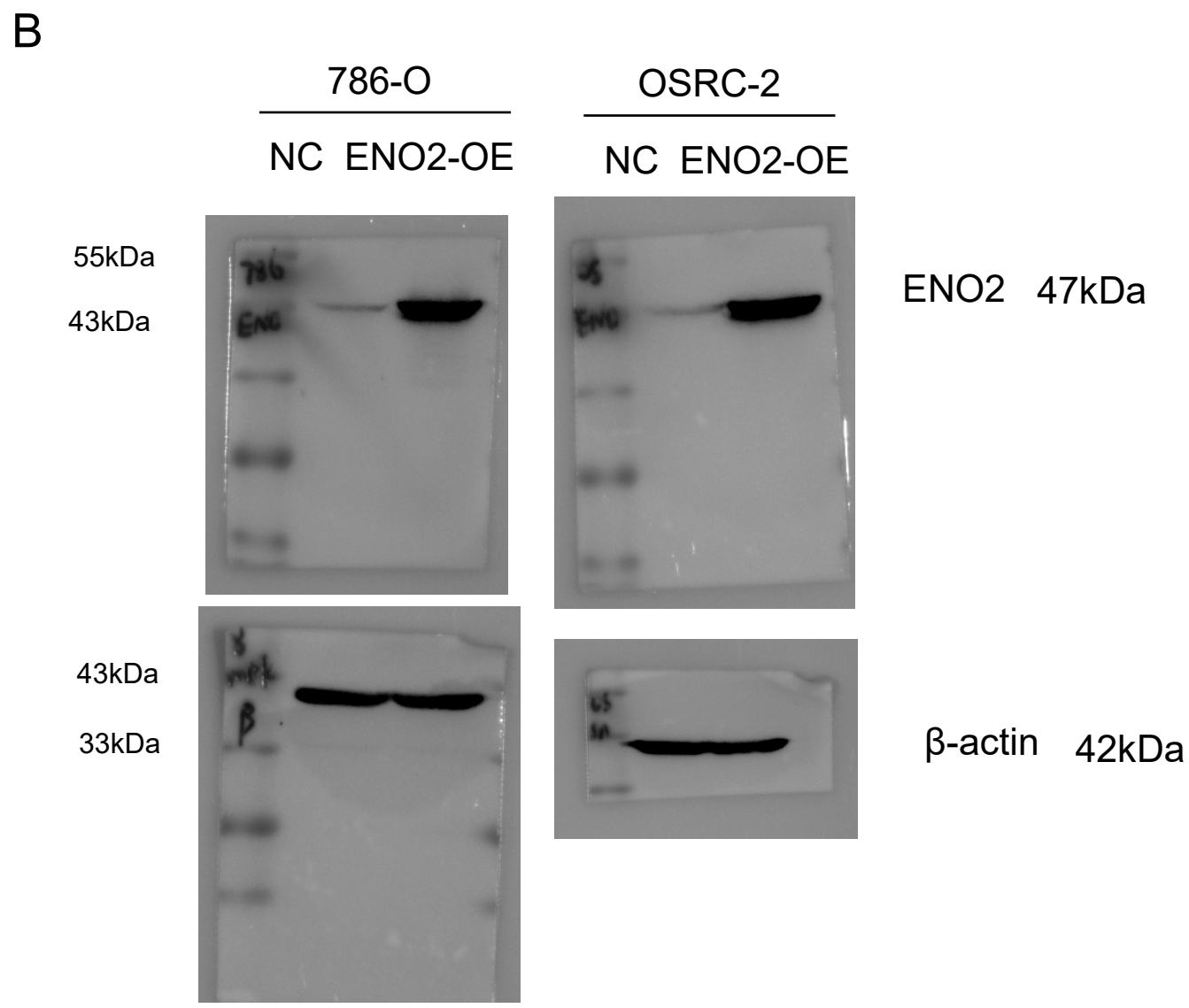
B
786-O
OSRC-2
NC
ENO2-OE
NC
ENO2-OE
55kDa
43kDa
ENO2
47kDa
43kDa
33kDa
β-actin
42kDa
